# Model-free decision making is prioritized when learning to avoid harming others

**DOI:** 10.1073/pnas.2010890117

**Published:** 2020-10-14

**Authors:** Patricia L. Lockwood, Miriam C. Klein-Flügge, Ayat Abdurahman, Molly J. Crockett

**Affiliations:** ^a^Department of Experimental Psychology, University of Oxford, Oxford OX1 3PH, United Kingdom;; ^b^Wellcome Centre for Integrative Neuroimaging, Department of Experimental Psychology, University of Oxford, Oxford OX3 9DU, United Kingdom;; ^c^Centre for Human Brain Health, School of Psychology, University of Birmingham, Birmingham B15 2TT, United Kingdom;; ^d^Department of Psychology, Yale University, New Haven, CT 06511

**Keywords:** moral, learning, model-free, prediction error, neuroimaging

## Abstract

Moral behavior requires learning how our actions help or harm others. Theoretical accounts of learning propose a key division between “model-free” algorithms that cache outcome values in actions and “model-based” algorithms that map actions to outcomes. Here, we tested the engagement of these mechanisms and their neural basis as participants learned to avoid painful electric shocks for themselves and a stranger. We found that model-free decision making was prioritized when learning to avoid harming others compared to oneself. Model-free prediction errors for others relative to self were tracked in the thalamus/caudate. At the time of choice, neural activity consistent with model-free moral learning was observed in subgenual anterior cingulate cortex (sgACC), and switching after harming others was associated with stronger connectivity between sgACC and dorsolateral prefrontal cortex. Finally, model-free moral learning varied with individual differences in moral judgment. Our findings suggest moral learning favors efficiency over flexibility and is underpinned by specific neural mechanisms.

## Significance

“Do no harm” is a universal principle of human social life. But how do we learn which of our actions help or harm others? Learning theory suggests there are two different systems that govern how we link actions and outcomes: a model-free system that is efficient and a model-based system that is deliberative. Here we show that people rely more on model-free decision making when learning to avoid harming others compared to themselves. Model-free neural signals that distinguish self and other are observed in the thalamus/caudate, and reliance on model-free moral learning for others varies with individual differences in moral judgment. These findings suggest that moral decision making for others is more model-free and has a specific neural signature.

A central component of human morality is a prohibition against harming others (1, 2). People readily avoid actions that might harm another person (3–7), and this basic harm aversion is so strong that many people even find it distressing to perform pretend harmful actions, such as shooting someone with a fake gun (8). Harm aversion is disrupted in clinical disorders such as psychopathy that have a strong developmental component (9), and although harm aversion is robust in healthy adults, anyone who has watched young children fighting over a coveted toy knows that such an aversion is not present from birth. Indeed, a large literature documents the emergence of moral conduct over the course of development (7, 10, 11). Cross-cultural differences in morality suggest moral behavior is fine-tuned to local environmental demands (12), and laboratory experiments demonstrate how individuals can quickly adapt moral behavior to changing norms (13, 14). All this evidence highlights a critical role for learning in the development of harm aversion and moral behavior more broadly (6). Once having learned as children that harming others is morally wrong, adults still need to learn which actions to take to avoid harm in novel contexts.

Recent work in computational neuroscience has advanced our knowledge of how organisms learn the value of actions and outcomes via reward and punishment (15, 16). An important theoretical distinction has been made between “model-based” and “model-free” learning systems (17, 18). Model-based learning is often described as deliberative learning, whereas model-free learning is thought to be habitual. The model-based system builds a “world model” of the environment and selects actions by prospectively searching the model for the best course of action (19, 20). In contrast, the computationally efficient model-free system assigns values to actions simply through trial and error. The distinction between these systems can be illustrated by giving the example of how we navigate home from work. The model-based system could easily replan if a particular route home was unexpectedly blocked, whereas a purely model-free learner can only plan a route home by directly experiencing each of the different routes (21). These two systems are also somewhat neurally dissociable, with model-based learning preferentially engaging lateral prefrontal cortex (LPFC), posterior parietal cortex, and caudate (20, 22, 23) and model-free learning preferentially engaging putamen (24, 25), although both systems update their representations via prediction errors encoded in overlapping regions of ventral striatum (20). Model-based and model-free systems often make similar recommendations about which actions are more valuable, but when they conflict an arbitration process allocates control between them (12, 13, 23, 26, 27). However, despite extensive theorizing that the model-based/model-free distinction may help to characterize puzzling features of moral learning and decision making (3, 28–30), it remains unknown whether the moral consequences of actions affect the balance between model-based and model-free control, and whether common or distinct neural processes are engaged when learning to avoid harmful outcomes to self and others.

Past work on the neural basis of moral decision making provides support for competing hypotheses. On the one hand, the sophistication of human morality seems to demand the kinds of complex representations afforded by model-based learning, suggesting learning to avoid harming others may preferentially engage the model-based system. Supporting this view, people are easily able to learn to avoid harmful actions without directly experiencing their outcomes, in line with a model-based learning strategy when avoiding harm to others (3, 28, 31). Moreover, moral decision making in healthy adults consistently engages brain regions most strongly associated with the model-based system, including LPFC, caudate, and temporoparietal junction (TPJ) (24, 26, 32). Deciding to follow moral norms like fairness and honesty, and enforcing those norms on others via costly punishment, engages LPFC (33–38), and disrupting LPFC function reduces moral norm compliance and enforcement (39, 40). During decisions to avoid harming others, LPFC encodes the blameworthiness of harmful choices and modulates action values in caudate and thalamus (4), two subcortical areas shown to play a critical role in associative learning and pain processing as well as moral decision making (41–46).

On the other hand, one principal function of model-free learning is to cache value in actions that are reliably adaptive, sacrificing flexibility for efficiency. Given that harming others is typically prohibited, actions that harm others may represent a special class of actions that are prioritized for model-free learning, similar to how certain classes of stimuli, like snakes and spiders, are “prepared” for aversive classical conditioning (47). In other words, since avoiding harm to others is hugely important for social life, learning processes that fast-track harm-avoidant action selection to a habitual, automatic process may be socially adaptive. Supporting this view, recent work suggests that morality constrains mental representations of what actions are considered possible; harmful actions are removed from choice sets as a default (48), and choices that harm others are slower than helpful choices, suggesting an automatic tendency to avoid harm (5, 49–51). Furthermore, recent studies of model-free learning to gain rewards for oneself and others have highlighted a distinct encoding of prediction errors concerning others’ outcomes in the subgenual anterior cingulate cortex (sgACC) (52, 53), a region that has been implicated in social and moral decision making more broadly (53–57). Model-free processes that distinguish learning about how one’s actions affect others could provide a neural mechanism for prioritizing model-free learning in moral contexts.

**Fig. 1.**
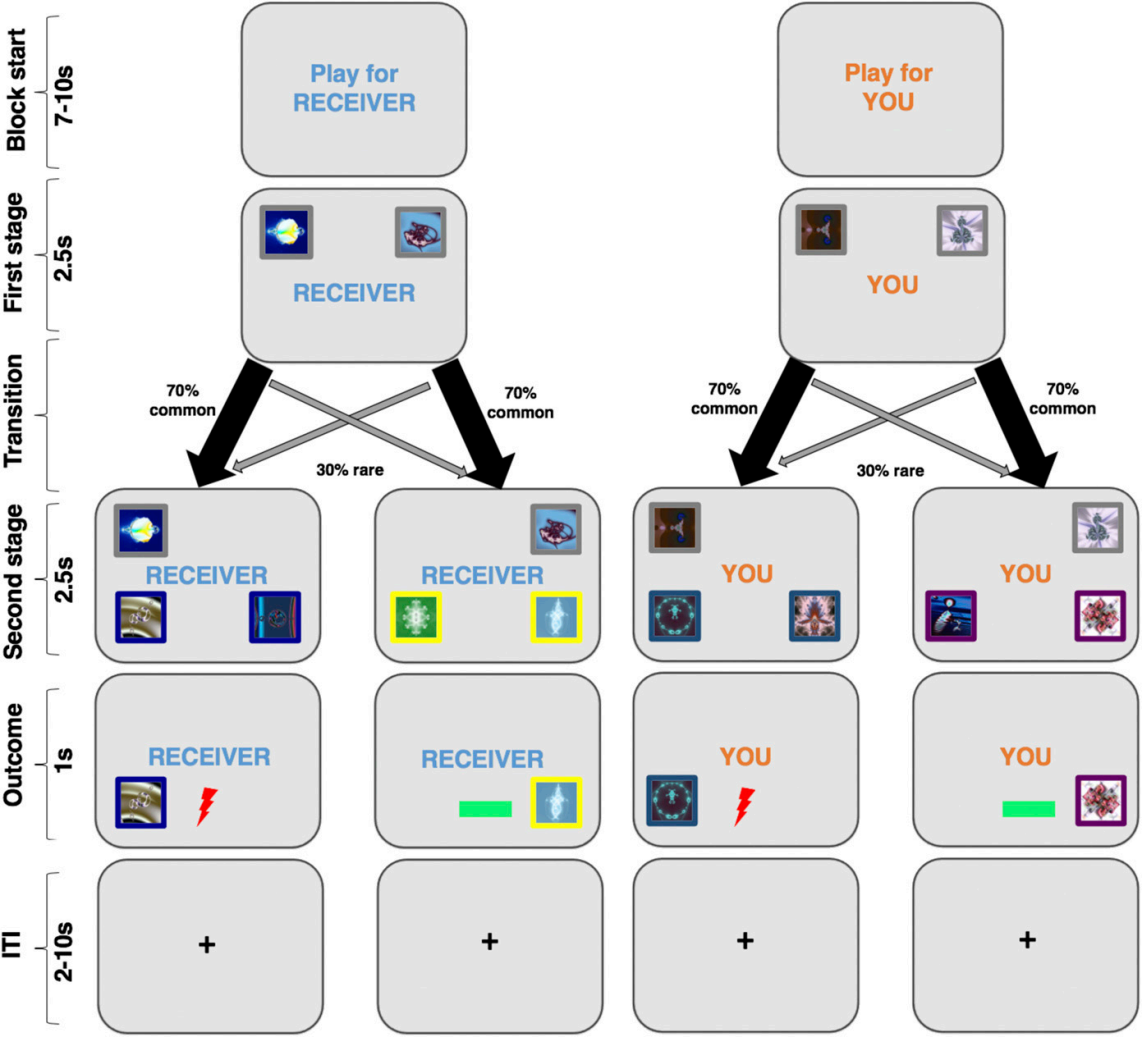
Model-free and model-based aversive learning task. Participants completed a two-stage decision-making task to assess the tendency to engage in model-free and model-based learning. The task was a hybrid of two tasks previously shown to assess model-free and model-based learning processes (20, 27). We used this task to probe learning to avoid aversive (shock) outcomes for either oneself or another person (the “receiver,” referred to as “other” hereafter). At the beginning of each block, an instruction cue signaled the recipient of the outcome (self or other). At the first stage, two images were displayed that probabilistically led to one of two states (common [∼70% of the time] or uncommon [rare] transition [∼30% of the time]), depicted by different colors surrounding the boxes. In this example, to “blue zone” or “yellow zone” for the other participant and “turquoise zone” or “purple zone” for self. Participants then made a second choice between two pictures in the colored zone which was followed by an outcome of shock or no shock. The probability with which the boxes at the second stage delivered a shock or no-shock outcome drifted throughout the experiment (bounded between 0 and 1 with a drift rate of 0.2) and participants were instructed to keep learning throughout. Ten percent of the total electric shocks accumulated in the “self” condition were delivered to the participant themselves at the end of the experiment, while 10% of the electric shocks accumulated in the “other” condition were delivered to the partner participant.

To test these competing hypotheses, we used computational modeling and functional MRI (fMRI) to probe the relative balance between model-based vs. model-free processes, and their neural bases, when people learn to avoid moderately painful electric shocks for themselves and a stranger. Forty-one participants attended a 3.5-h experimental session. After undergoing an extensive pain thresholding procedure (*Methods*), they completed a hybrid version of two paradigms previously proposed to reliably dissociate model-free vs. model-based learning (Fig. 1) (20, 23, 32). We optimized the task in a way that allowed us to address the specific hypotheses examined in the present study (see *SI Appendix*, *Supplementary Text* for details) and included as many as 272 trials per participant to accurately sample decisions for both self and other. Our final analysis included 36 participants who made a total of 9,792 choices.

## Results

### Model-Free Decision Making Is Prioritized When Learning to Avoid Harming Others vs. Self.

Participants completed a two-step decision-making task to index model-free and model-based learning strategies (Fig. 1). Prior to scanning, participants were trained on the transition structure of the task using stimuli different from the main experiment which allowed them to learn the probabilistic transition structure. The two-step task distinguishes model-free and model-based learning by measuring people’s choices to stay or switch based on the outcome (in this case pain or no pain) of the previous trial and the transition structure of the task (whether the trial was “common” or “rare”). On common trials (70%) participants’ choice at the first stage always leads to the same second-stage environment. On rare trials (30%), participants’ first-stage choice unexpectedly leads them to the opposite environment. Theoretically, a purely model-free learner would ignore the transition structure and repeat first-stage choices if they prevented pain on the previous trial but switch choices if the previous choice caused pain. Thus, model-free learning is reflected in a main effect of outcome (pain vs. no pain) on subsequent first-stage choice behavior. Participants switch after pain and stay after no pain. In contrast, a model-based learner would take the transition structure into account. While behavior on common transitions would be the same as for a model-free agent, after rare transitions a model-based agent would repeat a first-stage choice if the outcome was pain but switch if the outcome was no pain. Thus, model-based learning is reflected in an interaction between outcome (pain vs. no pain) and transition (common vs. rare). It is now well established that people display a combination of model-free and model-based behaviors when learning about rewarding outcomes (20, 32, 58), and initial evidence indicates that the same is true for learning about aversive (painful) outcomes for oneself (59, 60). We therefore first examined whether participants displayed a combination of model-free and model-based processes during aversive learning for oneself and others, as observed in these previous studies of reward learning. We conducted a series of model-agnostic and model-derived analyses where we sought to reproduce the same behavioral effect across different analytic approaches.

**Fig. 2.**
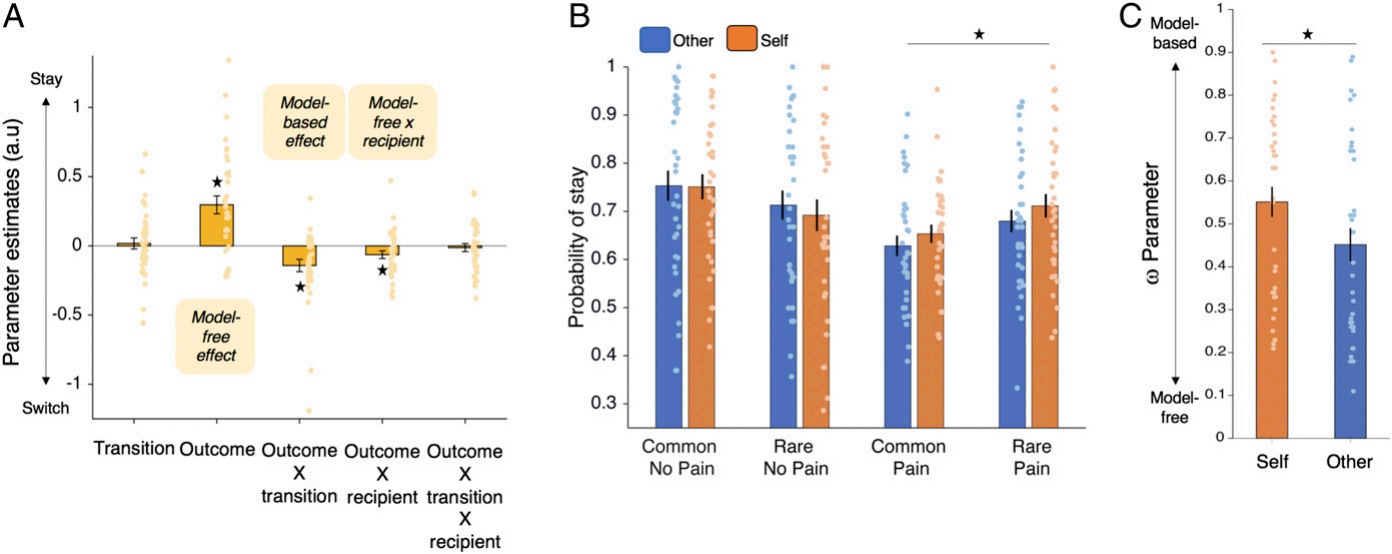
Model-free and model-based choices when avoiding harming oneself and others. (*A*) Logistic regression coefficients predicting first-level stay/switch choices (mean ± SEM). Participants exhibited a main effect of staying after no pain, which indicated model-free behavior [*t*(35) = 4.618, *P* < 0.001, CI for beta estimate: 0.17, 0.42, Cohen’s *d* = 0.77] and a outcome × transition interaction, which indicated model-based behavior [*t*(35) = −3.173, *P* = 0.003, CI for beta estimate: −0.23, −0.05, *d* = −0.53]. Intriguingly there was also an outcome × recipient interaction [*t*(35) = −2.31, *P* = 0.027, CI of beta estimates −0.12, −0.008, *d* = −0.39] showing that participants were more model-free, and thus more likely to switch after pain and stay after no pain, independent of transition type, when making choices for another person. (*B*). The probability of repeating a choice at the first level (“stay”) is plotted as a function of the transition and outcome on the previous trial. This shows that the outcome × recipient interaction in *A* is mostly driven by fewer stay trials after pain, regardless of transition (two rightmost blue vs. orange bars). Thus, the more pronounced model free behavior for others is mostly driven by a lower probability of staying after pain outcomes rather than a higher probability of staying after no pain outcomes). (*C*) ω estimates from the best-fitting model showed that the ω parameter was significantly lower for other (0.45) than self (0.55), consistent with the regression analyses that showed people were more model-free when avoiding harm to others compared to self (*P* < 0.02). Asterisks indicate significant difference at *P* < 0.05.

To parallel previous work on model-free and model-based behavior (20), we began our analyses by using a logistic regression to assess model-free and model-based behavior and interactions with recipient, before showing that these results were robust to including random effects. We predicted first-stage stay vs. switch choices as a function of the outcome (pain or no pain), transition on the previous trial (common or rare), and recipient (self vs. other). Specifically, we built a model that tested whether people showed model-free behavior, model-based behavior, and an effect of recipient either interacting with the model-free and/or the model-based component. We found a significant main effect of outcome [*t*(35) = 4.618, *P* < 0.001, CI for beta estimate: 0.17, 0.42, Cohen’s *d* = 0.77] indicating a contribution of model-free learning but also a significant transition by outcome interaction [*t*(35) = −3.173, *P* = 0.003, CI for beta estimate: −0.23, −0.05, *d* = −0.53], indicating the presence of model-based learning (Fig. 2 *A* and *B*). These findings demonstrate that, similar to reward learning, aversive learning is underpinned by a mixture of model-based and model-free processes, regardless of whether outcomes are for oneself or another person.

Despite evidence of both model-free and model-based learning strategies, it could be the case that one strategy is particularly prioritized when learning to avoid harm to self or other. To test this, we examined interactions between shock recipient and the model-free and model-based components of learning. We found a significant interaction between outcome (the model-free contribution to learning) and recipient, showing that people were more model-free for others relative to self [*t*(35) = −2.31, *P* = 0.027, CI of beta estimates −0.12, −0.008, *d* = −0.39]. Importantly, there was no interaction between transition × outcome (the model-based component of learning) and recipient [*t*(35) = −0.459, *P* > 0.64, CI of beta estimates: −0.071, 0.045, *d* = −0.08, BF_01_ = 5.1, providing substantial evidence in support of the null].

Because model-based behavior may in some cases be more effortful but does not achieve better outcomes in this version of the task, it is possible that a model-based learner might learn to become more model-free over the course of the task. We therefore also assessed whether our estimate of being model-free interacted with trial number. However, no significant association was observed [*t*(35) = −0.88, *P* = 0.39, CI for beta estimate: −0.11 0.05, BF_01_ = 3.9, providing substantial evidence in support of the null]. For additional robustness, we also assessed whether our effect of being more model-free for other changed over the course of the experiment. Again the interaction of outcome, recipient, and trial number was not significant [*t*(35) = −0.05, *P* = 0.74 CI for beta estimate: −0.05 0.07, BF_01_ = 5.3, providing substantial evidence in support of the null], speaking against the possibility that participants learned to become more model-free for others but not self across the experiment.

We next validated these behavioral results by repeating our analysis using linear-mixed effects models with the lme4 package in R, which ensured we had good estimates of random effects and accounted for variability in behavior using Bound Optimization by Quadratic Approximation (*Methods*). We included all main effects and interactions, and random slopes, and found all results remained the same (*SI Appendix*, *Supplementary Text*). Taken together, these findings support the idea that people were more model-free when avoiding harming others but not less model-based.

To further examine which outcomes most influenced the outcome × recipient interaction, we performed separate post hoc tests on the percentage of stay/switch choices for self and other following: 1) only pain outcomes and 2) only no-pain outcomes. This showed that the recipient difference was driven by increased switching after pain outcomes for other vs. self (*P* = 0.028, Cohen’s *d* = 0.38, 95% CI = [0.041, 0.72]), with no statistically significant difference between recipients in the proportion of stay/switch choices after no pain outcomes (*P* = 0.552, *d* = 0.10, 95% CI = [−0.23, 0.43], BF_01_ = 4.72 providing substantial evidence in support of the null). This is important because the specificity of the effect rules out that people were simply more indifferent or inattentive to the outcomes of others compared to self.

Finally, to confirm that our findings were consistent with a harm-aversion account rather than an effort or inattention account, we examined reaction times in the self and other conditions, with the idea that differences in reaction times might index greater inattention or cognitive effort for self vs. other trials. There was no difference in reaction times (RTs) overall when deciding for self compared to other [*F*(1,35) = 0.17, *P* = 0.69, η^2^ = 0.05, BF_01_ = 6.87, substantial evidence in support of the null], although as expected participants were slower to make decisions to switch relative to stay in general [*F*(1,35) = 11.73, *P* = 0.002, η^2^ = 0.25].

To summarize, choice data provided clear evidence for more pronounced model-free behavior for other compared to self and were not consistent with merely showing less model-based or more random behavior. This is because participants specifically switched more after causing harmful outcomes to others, independent of transition type, and there were no recipient differences in RT latencies.

### Computational Modeling of Aversive Learning for Self and Other.

Next, we fitted several trial-by-trial computational models to our data to examine further which model best captured the described behaviors during aversive learning for self and other. Deriving such trial-by-trial estimates that capture individual choice preferences was a prerequisite for modeling the fMRI data and allowed us to support our logistic regression analyses. We started with the full seven-parameter model proposed by Daw et al. (20) and compared this model to similar models with fewer parameters (four or five) following modifications similar to those suggested in previous studies (e.g., refs. 61 and 62; for details, see *Methods* and *SI Appendix*, Table S2 and *Supplementary Text*). We also included variants of the same models that involved separate learning rates for pain and no-pain outcomes, given evidence suggesting differential learning as a function of outcome valence (e.g. refs. 63 and 64). All of these models were initially fitted separately on self and other blocks.

We found that a five-parameter model best explained behavior compared to all alternative models tested. This model included separate learning rates for no pain and pain outcomes (αPain, αNoPain), a single temperature parameter capturing choice randomness (β), a perseverance parameter capturing a tendency to stick with the previously made choice (ρ), and a model-free/model-based weighting parameter (ω). Importantly, this five-parameter model best explained behavior in both the self and other blocks (*SI Appendix*, Table S1).

We next compared the different estimated parameters for the self and other blocks. This analysis showed a significant difference between the conditions in both the perseverance parameter ρ (*t*(35) = 2.41, *P* = 0.02, *d* = 0.40, 95% CI = [0.06, 0.74]) and the model-free/based weighting parameter ω (*t*(35) = 3.10, *P* = 0.0039, *d* = 0.51, 95% CI = [0.16, 0.86]; *SI Appendix*, Table S2). To compare these parameters more robustly, we then used maximum a posteriori estimation performed on the pooled data of self and other blocks to compare three models, one with separate perseverance ρ and ω parameters for self and other (and thus a total of seven parameters), one with separate ω parameters for self and other (and therefore a total of six parameters), and the original five-parameter model (which assumes the same ρ and ω across self and other blocks). We used these models to examine whether differences in the ρ and ω parameters between self and other when fitted separately reflected true differences in the weighting of these parameters in a model comparison. This analysis showed that the model with separate ω’s for self and other, but not separate ρ’s, best explained the data. Importantly, these ω parameters were also significantly different from one another (selfω = 0.55, otherω = 0.45, *t*(35) = 2.41, *P* = 0.02, *d* = 0.40, 95% CI = [0.06, 0.74]; Fig. 2*C* and *SI Appendix*, Tables S3 and S4). Thus, consistent with the regression-based behavioral analyses that did not rely on a computational model (Fig. 2*A*), participants were more model-free than model-based when learning to avoid harming others, compared to self (Fig. 2*C*). See *Methods* and *SI Appendix*, *Supplementary Text* for further details.

Finally, we ran several control analyses. First, we tested whether any participants might be actively trying to harm the other person by allowing the beta parameter to take on negative values (although we note that a negative beta might also reflect poorer learning rather than willingness to inflict pain on others). This analysis showed no participant was given a negative beta weight. Second, recent evidence suggests that model-free behavior might reflect the use of different or nonstandard models and can reflect poorer model fits (65). We therefore also assessed whether there were differences in model fits between the self and other conditions. We compared the negative log-likelihood, which is the sum of the logarithm of the choice probabilities and the measure of the fitting error that is minimized during fitting. Comparison of the individual negative log-likelihoods for self vs. other was not significant [*t*(35) = 0.18, *P* = 0.86; BF_01_ = 5.5, substantial evidence for the null].

Next, we confirmed that participants were not incentivized to employ a more model-free or model-based strategy. We ran a simulation of purely model-free (ω = 0) and purely model-based (ω = 1) agents. Comparing the total shocks accumulated for these two types of agents showed no significant difference [*t*(198) = 0.45, *P* = 0.649)] and Bayesian analysis showed substantial evidence in support of the null (BF_01_ = 5.90). We then compared the total shocks accumulated in the self and other conditions from our participants and again showed no significant difference [*t*(35) = −0.211, *P* = 0.834)] with Bayesian analyses providing substantial evidence for no difference (BF_01_ = 5.47).

Together, these analyses confirm that our results were not related to differences in model fits, there were no participants who were trying to actively harm others, and that, as we intended, our modified two-step task did not incentivize a model-free or model-based strategy (see also *SI Appendix*, *Experimental Note in Introduction*).

### Subcortical Areas Distinguish Model-Free Prediction Errors for Self and Other.

Previous neuroimaging studies of model-based and model-free reward learning have reported model-free prediction error signals in ventral striatum (20). We therefore first sought to replicate this effect in our aversive learning paradigm. To facilitate comparison with previous studies of reward learning, no-pain outcomes were coded as 1 and pain outcomes coded as 0. Therefore, a positive prediction error represents unexpected pain relief/avoidance, and a negative prediction error represents unexpected pain.

**Fig. 3.**
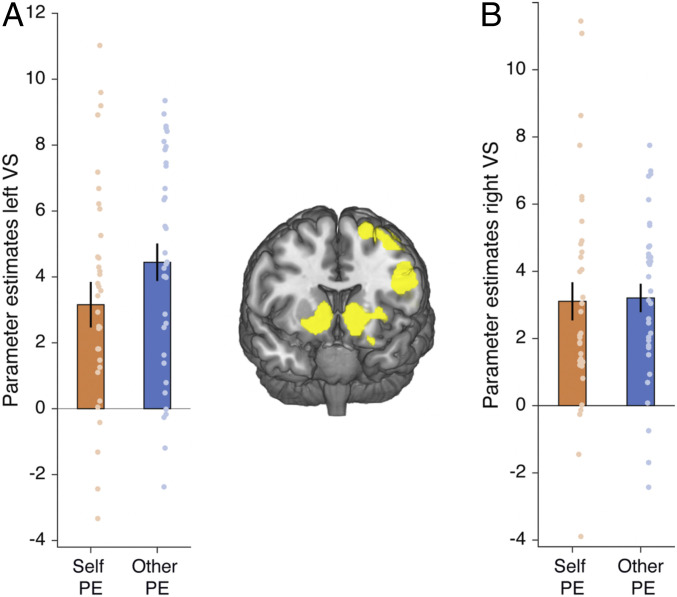
(*A* and *B*) Ventral striatum (VS) encodes prediction errors (PE) of pain avoidance for self and other. Ventral striatum (right x = 10, y = 12, z = −4, k = 236, z = 5.84; left x = −16, y = 6, z = −10, k = 458, z = 5.77, *P* < 0.05 FWE whole brain-corrected after initial thresholding at *P* < 0.001) tracked model-free prediction errors for both self and other bilaterally, with no significant differences between conditions.

**Fig. 4.**
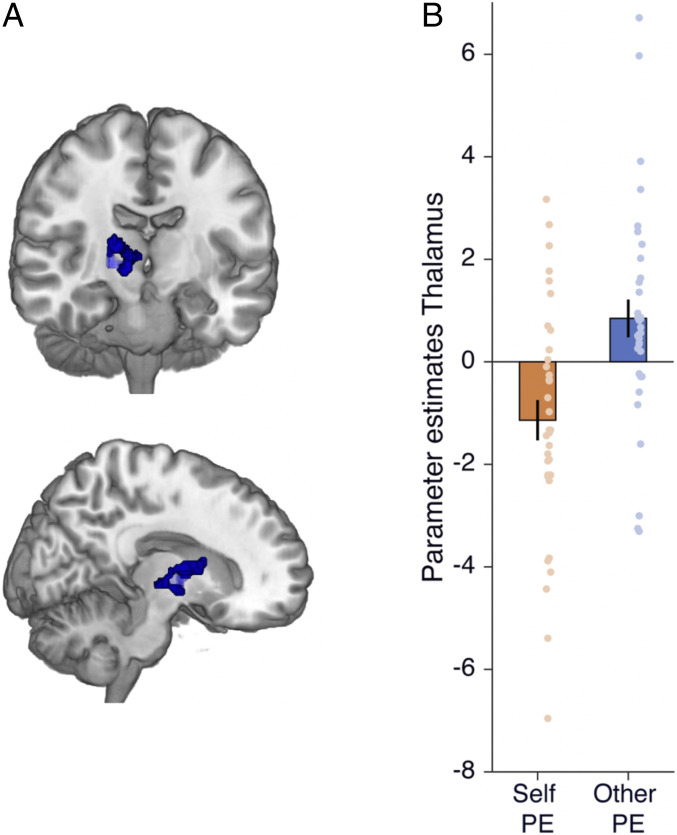
Thalamus/caudate signal distinguishes model-free prediction errors for avoiding harm to other vs. self. (*A*) Thalamus cluster from the contrast other prediction error > self prediction error (x = 16, y = −18, z = 0, k = 84, *P* = 0.033, Z = 3.50 FWE-SVC from an independent anatomical mask of the thalamus and x = 12 y = −2 z = 4, Z = 4.08, k = 125, *P* = 0.005 FWE-SVC after initial thresholding at *P* < 0.001 for a functional ROI derived from Neurosynth to the term “pain” and x = 10 y = −4 z = 4, Z = 3.83, k = 54, and *P* = 0.002 FWE-SVC for an 8-mm sphere from a meta-analysis of observed pain (68) overlaid on an anatomical scan to show the extent of activation. (*B*) For illustration, parameter estimates extracted from the thalamus cluster are shown separately for self and other prediction error (PE). See *SI Appendix*, Fig. S1 for a meta-analysis of neuroimaging studies with the term “pain” that highlights overlapping activation in the thalamus.

**Fig. 5.**
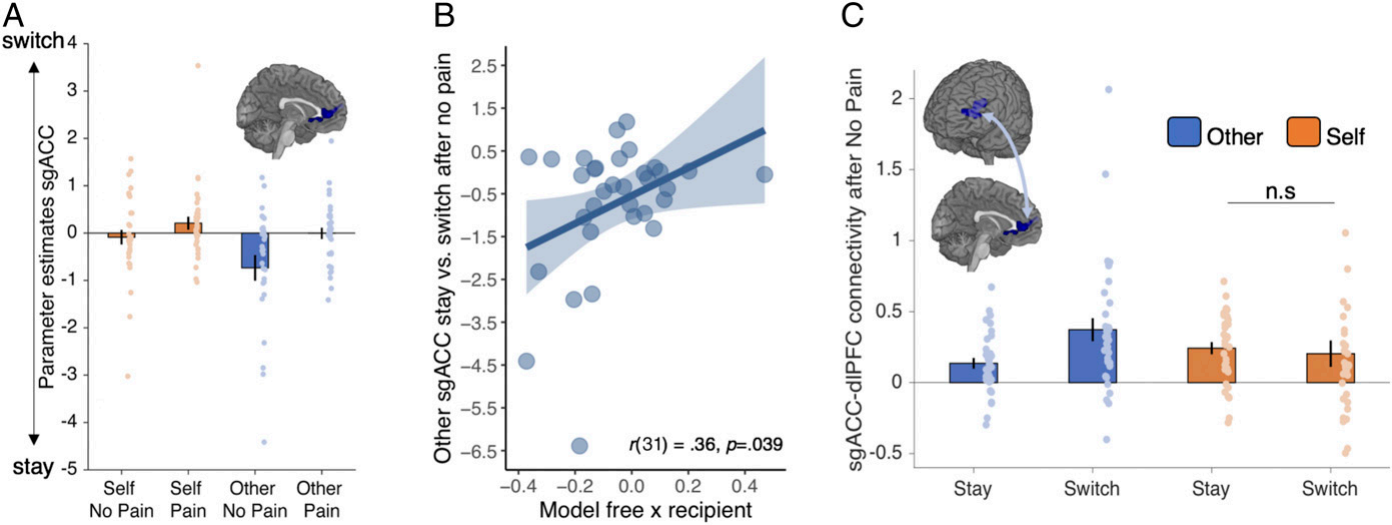
sgACC tracks stay vs. switch after no pain for other and connects more strongly to dlPFC when switching after no pain for other. (*A*) sgACC response (x = −2, y = 36, z = 6, K = 498, *P* = 0.028, FWE whole brain-corrected, after initial thresholding at *P* < 0.001) to stay vs. switch after no pain for other overlaid on the medial surface of an anatomical scan. The observed BOLD pattern is consistent with model-free behavior (*B*). Bivariate association between parameter estimates for stay vs. switch after no pain for other in sgACC and greater model-free behavior for other (more negative on *x* means relatively more model-free for other compared to self). (*C*) sgACC cluster connects to dlPFC (x = −46, y = 38, z = 26, k = 382, Z = 4.12, *P* = 0.039, FWE whole brain-corrected after initial thresholding at *P* < 0.001) during decisions to switch relative to stay after no pain for other. Average slope estimates across participants show stronger connectivity during switch decisions than stay decisions after receiving no pain for other (blue bars) but no difference in sgACC–dlPFC connectivity between stay and switch for self [*t*(32) = 0.43, *P* = 0.667, *d* = 0.08, 95% CI for Cohen’s *d* = −0.27 0.42; BF_01_ = 4.92 providing substantial evidence in support of the null] (orange bars). n.s, not statistically significant.

We built a general linear model (GLM1) that contained onsets for the first-stage choice, second-stage choice, and outcome separately for self and other trials. These three time periods were each associated with parametric modulators from our winning model. These included the value difference between the two options at the first-stage choice, the state prediction error based on the transition at the second-stage choice, and the model-free prediction error at the time of the outcome. We focused our analysis on model-free prediction errors at the time of the outcome for two reasons. First, our behavioral effects showed that self/other differences in learning emerged for model-free but not model-based learning. Second, model-free and model-based prediction errors are highly correlated and careful examination of their separate influences has shown that they are both encoded in ventral striatum (20). Significant activations are overlaid on anatomical images using the MRIcron software for Figs. 3–5. Following standard procedures for multiple comparisons correction in fMRI, main effects are reported at *P* < 0.05, familywise error (FWE) cluster-corrected across the whole brain after initial thresholding at *P* < 0.001, or *P* < 0.05 FWE small volume-corrected (SVC) after initial thresholding at *P* < 0.001 for a priori regions of interest (ROIs) (66, 67).

We began our analyses by examining whether previously reported neural correlates of value difference and state prediction errors were also observed in our paradigm. Several areas tracked inverse value difference and thus showed larger responses for choices that had a smaller value difference between the two first-stage options, including the most dorsal parts of anterior cingulate cortex near pre-supplementary motor area, bilateral inferior parietal cortex, and middle frontal gyrus (*SI Appendix*, Table S5), regions previously associated with the tracking of inverse subjective value difference (69–71). These signals did not differ between self and other (*SI Appendix*, Table S5). Also consistent with previous findings (22), we found evidence of a main effect for state prediction errors at the second stage in dorsal ACC (x = −6, y = 10, z = 52, Z = 4.85, K = 906, *P* < 0.001 FWE-corrected) that again showed overlap between self and other (*SI Appendix*, Table S5).

Next, we tested whether model-free prediction errors were present in ventral striatum, as reported in a previous study examining model-based and model-free reward learning for self (20) and several studies of reward-based reinforcement learning (72). We found a large bilateral cluster signaling prediction errors of harm avoidance (positive for no pain, negative for pain) in ventral striatum (right x = 10, y = 12, z = −4, k = 236, Z = 5.84; left x = −16, y = 6, z = −10, k = 458, z = 5.77, *P* < 0.05 FWE whole brain-corrected after initial thresholding at *P* < 0.001; Fig. 3). Again, this signal did not significantly differ for self and other conditions [right *t*(34) = −0.14, *P* = 0.89, BF_01_ = 5.4, providing substantial evidence in support of the null; left *t*(34) = −1.47, *P* = 0.152, BF_01_ = 4.0, providing substantial evidence in support of the null].

Given that our behavioral results indicated that model-free decision making was prioritized when learning to avoid harm to others (relative to self), we next sought to identify areas that distinguished model-free prediction errors for others (relative to self). Importantly, our paradigm was designed to specifically assess how people learn to avoid harming self and other, rather than how they benefit self and other by collecting rewards, as in previous studies (20). We therefore focused our analyses on areas of the brain that have previously been linked to pain processing and aversive learning, as well as those involved in learning in general and social decision making (*Methods*).

This analysis revealed a cluster in the thalamus extending into the caudate [x = 16, y = −18, z =0, k = 84, *P* = 0.033, Z = 3.50 FWE-SVC from an independent anatomical mask of the thalamus and x = 12, y = −2, z = 4, Z = 4.08, k = 125, *P* = 0.005 FWE-SVC after initial thresholding at *P* < 0.001 for a functional ROI derived from Neurosynth to the term “pain” and x = 10, y = −4, z = 4, Z = 3.83, k = 54, and *P* = 0.002 FWE-SVC for an 8-mm sphere from a meta-analysis of observed pain (68); Fig. 4 *A* and *B* and *SI Appendix*, Fig. S1]. The cluster positively tracked prediction errors of pain avoidance when learning for other (*t*(33) = 2.30, *P* = 0.028, Cohen’s d = 0.39, 95% CI = [0.04, 0.74]) and negatively tracked prediction errors of pain avoidance when learning for self (*t*(33) = −2.89, *P* = 0.007, Cohen’s d = −0.50, 95% CI = [−0.85, −0.14]). Although this cluster extended into the caudate, the caudate ROI itself was not significant (x = 18, y = 6, z = 0, Z = 3.49, k = 2, *P* = 0.064, FWE-SVC after initial thresholding at *P* < 0.001) (see *SI Appendix*, Table S6 and *Supplementary Text* for additional Bayesian first-level analyses comparing prediction error and outcome only models of neural response).

### Signals Consistent with Model-Free Influence Are Encoded in sgACC.

One signature of model-free learning is a tendency to repeat previously rewarded actions and avoid previously punished actions, regardless of experienced transitions (20). Such a model-free influence is thought to emerge at the time of choice by activating the reinforcement histories of potential actions and driving selection of the most valuable action in terms of its recent history (73). In the context of our task, through model-free influence, an action that was unpunished on the previous trial should be prioritized for selection (“stay”), while an action that was punished on the previous trial should be avoided (“switch”). Importantly, because model-free learning is insensitive to task structure, this process should occur regardless of whether the transition from the first to the second stage experienced on the previous trial was common or rare.

Therefore, to probe the neural signatures consistent with a model-free influence at the time of choice, and any potential differences between self and other conditions, we examined neural responses during “switch” and “stay” choices at the first stage as a function of the outcome on the previous trial (no pain or pain). We created an additional GLM (GLM2) that modeled the onset of self trials after pain, self trials after no pain, other trials after pain, and other trials after no pain, with stay (−1) and switch (1) coded as parametric modulators of each of these onsets. Thus, our analysis examined differential neural encoding of stay vs. switch decisions on the current trial, as a function of the outcome on the previous trial (pain or no pain) and its recipient (self or other).

Our analysis revealed a signal in sgACC consistent with a model-free influence on “other” trials (x = −2, y = 36, z = 6, K = 498; Z = 3.88, *P* = 0.028, FWE whole brain-corrected). This region was more active during stay relative to switch choices on the current trial, following a “no pain” outcome on the previous trial, selectively in the “other” condition (Fig. 5*A*). Corroborating the view that this signal reflects a model-free influence, responses in sgACC were positively associated with the model-free × recipient interaction in behavior (*r*(31) = 0.36, *P* = 0.039, 95% CI = [0.02, 0.62]; Fig. 5*B*) such that participants with the largest sgACC difference between stay and switch following no pain for other also showed the strongest prioritization of model-free learning for others relative to self (for results relating to our TPJ ROI, see *SI Appendix*, Fig. S2 and *Supplementary Text*). To provide further evidence that this signal was consistent with model-free processing, we conducted an additional confirmatory analysis (GLM3) where we modeled onsets separately for stay vs. switch choices with two parametric regressors—a model-free (outcome [no pain/pain]) and a model-based (outcome × transition [no pain/pain × common/rare transition]) regressor that competed for variance (correlations were all <0.4). This analysis showed overlapping voxels in sgACC compared to those identified in GLM2 that tracked the model-free but not the model-based regressor (*SI Appendix*, Fig. S3 and *Supplementary Text*). Finally, we confirmed using bootstrapping that the effect seen in sgACC after rare transitions was comparable to the effect observed after common transitions (*SI Appendix*, Fig. S4).

### Increased Functional Connectivity between sgACC and Dorsolateral Prefrontal Cortex When Switching after No Pain for Other.

Behavioral analyses indicated that participants on average showed a mixture of model-based and model-free strategies but prioritized model-free decision making when avoiding harm to others. Thus, we next sought to identify regions that might modulate the model-free effects observed specifically in the “other” condition in sgACC (Fig. 5*A*). We therefore conducted psychophysiological interaction (PPI) analyses (GLM4) to assess functional connectivity between sgACC and the whole brain as a consequence of staying vs. switching after no pain for other (see *Methods* for additional details of analyses). This analysis revealed that functional connectivity between sgACC and dorsolateral prefrontal cortex (dlPFC) was significantly different during switch vs. stay decisions following no pain for other (x = −46, y = 38, z = 26, k = 382, Z = 4.12, *P* = 0.039, FWE whole brain-corrected after initial thresholding at *P* < 0.001).

Because the PPI was performed on the difference between stay and switch trials, it did not give us insight into the sgACC–dlPFC coupling separately for stay and switch. Therefore, to further understand the nature of this effect we plotted the average slope of sgACC and dlPFC connectivity during stay and switch choices following no pain for other (Fig. 5*C*). This showed that there was positive coupling between sgACC and dlPFC for both stay and switch trials, with stronger positive coupling during switch choices compared to stay choices after receiving no pain for another person (Fig. 5*C*). We did not observe significant differential coupling between sgACC and dlPFC during switch compared to stay choices following no pain for self (GLM5; *t*(32) = 0.43, *P* = 0.667, *d* =0.08, 95% CI = [−0.27 0.42]; BF_01_ = 4.92 providing substantial evidence in support of the null; Fig. 5*C*). See *SI Appendix*, *Supplementary Text* for two additional GLMs (GLM 4.1 and 4.2) testing the profile of dlPFC response.

### Individual Differences in Moral Judgment Relate to Model-Free Moral Learning.

Finally, we conducted exploratory analyses to examine whether individual differences in moral judgment were related to individual differences in model-free moral learning and its neural basis. Theoretical work has suggested that model-free processes might explain tendencies to reject instrumentally harming others, even when instrumental harm results in better outcomes overall (3, 28). To test this, we measured individual differences in endorsement of instrumental harm using the instrumental harm component of the Oxford Utilitarianism Scale (74), which measures agreement with statements like “It is morally right to harm an innocent person if harming them is a necessary means to helping several other innocent people” and “Sometimes it is morally necessary for innocent people to die as collateral damage—if more people are saved overall.” Consistent with theoretical predictions, we found a significant positive relationship between rejection of instrumental harm and model-free learning for others, such that those people who most strongly rejected instrumental harm were the most model-free when avoiding harm to others (*r*(34) = 0.37, *P* = 0.026, 95% CI = [0.05, 0.62]; *SI Appendix*, Fig. S5). Furthermore, consistent with past work linking endorsement of instrumental harm with dlPFC function (75, 76), we found that endorsement of instrumental harm was positively correlated with dLPFC–sgACC connectivity when switching choices after causing harm to others (*r*(31) = 0.43, *P* = 0.012, 95% CI = [0.11, 0.68]; *SI Appendix*, Fig. S5).

Second, we probed the sensitivity of moral wrongness judgments to how much suffering an action inflicts on a victim (“outcome sensitivity”) vs. how aversive it feels to perform the action [“action sensitivity” (77)]. Past work has connected the former with model-based learning and the latter with model-free learning (3, 28). However, we note that model-free learning is directly sensitive to recent outcomes (18), which might lead to an association between model-free behavior and outcome sensitivity. Participants evaluated the moral wrongness of 23 harmful actions that varied independently in how much suffering they would cause vs. how aversive they would feel to perform. Action sensitivity and outcome sensitivity were inversely correlated (*r*(34) = −0.40, *P* = 0.016, 95% CI = [−0.08, −0.64]). Partial correlations controlling for action sensitivity revealed that outcome sensitivity was positively correlated with several aspects of model-free moral learning (*SI Appendix*, Fig. S6), including the tendency to switch following harm to others (*r*(33) = −0.37, *P* = 0.029, 95% CI = [−0.04, −0.62]), the strength of model-free prediction error signals for other vs. self in thalamus/caudate (*r*(31) = 0.385, *P* = 0.027, 95% CI = [0.05, 0.64]), and the strength of model-free influence in sgACC (*r*(30) = −0.374, *P* = 0.035, 95% CI = [−0.04, −0.64]).

The reverse partial correlations testing for the effect of action sensitivity while controlling for outcome sensitivity were not significant (all *P*’s > 0.08, all |r’s| < −0.30). Together these findings suggest that a natural tendency to engage in model-free moral learning when avoiding harm to others is related to how moral judgments of others’ harmful actions track with harm severity (see *SI Appendix*, *Supplementary Text* for correlations with the ω parameter).

## Discussion

Learning to avoid actions that harm other people is a fundamental prerequisite for moral behavior. Here we show that people prioritize model-free decision making when learning which actions have the potential to harm others and that learning to avoid harming others (vs. self) has a distinct neural signature. The thalamus/caudate differentially encoded prediction errors of pain avoidance for self vs. other, while sgACC positively tracked with model-free influence on pain avoidance at the time of choice. Overriding model-free influence when choices affected others invoked stronger connectivity between sgACC and dlPFC. Finally, multiple aspects of moral judgment were associated with model-free moral learning and its neural correlates.

In the context of our study, model-free moral learning manifested as a reduced likelihood of repeating actions that harmed others on the previous trial, regardless of whether such actions typically led to states with a high likelihood of harmful outcomes. Our behavioral finding that people were more model-free when learning to avoid harming others relative to themselves suggests that potentially harmful actions might be prioritized for automatic avoidance as a default. Given the importance of avoiding harm to others for social life, such a learning mechanism would be socially adaptive. Our findings are consistent with prior work showing that morality constrains mental representations of what actions are considered possible, with harmful actions removed from choice sets as a default stance (48). Repeatedly assigning negative action values to harmful actions over the course of one’s life might automatically remove such actions from consideration, even though in some cases locally harmful actions can lead to wider benefits (e.g., a surgeon cutting open a patient to remove a cancerous tumor). If such a learning strategy is socially adaptive, this raises interesting questions about whether model-free learning can be considered an “optimal” strategy in the ecological sense.

An alternative explanation for our behavioral findings is that model-based learning is effortful (27), and people choose to put in less effort to benefit others (78). Relatedly, after harming another person this might cause someone to become more distracted or more uncertain about the impact of the outcome, leading behavior to appear more model-free. With regards to the effort account, such an explanation seems unlikely given that model-free moral learning in our study was specifically driven by a lower probability of repeating choices that harmed others, while there was no difference between self and other on trials that avoided harm. For further discussion of the effort account, see *SI Appendix*, *Supplementary Discussion*. Finally, our results are also not likely to be attributable to differences in the subjective perception of the harmfulness or aversiveness of the outcomes for others compared to self, as participants overall rated shocks received for others in the task as being just as aversive as shocks received for themselves, and differences in shock aversiveness did not correlate with model-free behavior. Taken together these results suggest that people might naturally prioritize model-free decision making when learning to avoid harming others, and these effects are unlikely to be explained by less effort or engagement. However, these arguments do not completely rule out alternative explanations and future studies could use optimized designs to manipulate cognitive effort or social uncertainty to test how these influence model-free learning.

Recent work has suggested that apparently model-free behavior in the two-step task might reflect the use of a different model, rather than necessarily being model-free (65). We did not observe any difference in model fits between the self and other conditions, suggesting our observation of apparently greater model-free learning for other than self does not simply reflect a poorer model fit for other. However, our data show that participants were particularly sensitive to whether the outcomes were pain or no pain for other, which might be explained by having a slightly more complex model than being purely model-free in the strict sense.

Turning to the neural findings, we observed a signal in the thalamus, extending to the caudate, that differentially encoded model-free prediction errors when learning to avoid harming others vs. self. These subcortical regions were previously observed to encode value during moral decisions to avoid profiting from others’ pain (4) and play a critical role in associative learning and moral decision making more broadly (41, 43, 45, 46). The thalamus is often linked to the processing of the affective dimension of pain in addition to its sensory properties (79). For example, microstimulation of the thalamus can invoke affective memories of previously experienced pain (46). The thalamus/caudate signal differed from the adjacent ventral striatum response that positively tracked model-free prediction errors regardless of the recipient of the outcome, consistent with a previous study using a similar task with rewarding outcomes for self only (20). These findings suggest that multiple subcortical areas support model-free moral learning, perhaps with ventral striatum providing a generic model-free prediction error signal that is insensitive to outcome valence and outcome recipient, and thalamus/caudate providing additional information about social context.

Another signature consistent with model-free influence was observed in sgACC at the time of choice, contingent on the outcome of the previous trial and specific to the “other” condition. Specifically, signal in sgACC was higher when participants repeated actions that previously avoided harming others, but not during similar choices for oneself. Individual differences in model-free behavior also tracked with individual differences in sgACC response at the time of choice. Notably, previous work has implicated sgACC in model-free learning to gain rewards for others but not self (52) and in receiving unexpected positive feedback from others (80), suggesting this region might compute learning signals that are specific to social settings (81). More broadly, activity in sgACC has been positively associated with prosocial and moral behaviors (15, 53, 54, 56).

Collectively these findings suggest that sgACC might bias decision making away from choices that could harm others. In contrast, we found that when participants made a decision to switch their choice after causing harm to others, there was increased functional connectivity between sgACC and dlPFC. These two regions showed stronger coupling on trials where participants abandoned a choice that previously avoided pain for others, compared with trials where participants repeated actions that previously spared others from pain. Although we cannot confidently attribute these patterns to model-based control, past work has implicated dlPFC in model-based learning and decision making (32, 73). In parallel, research has linked these regions to the adjustment of moral decisions to blame and punishment (5, 33, 34, 40, 82).

Finally, we observed correspondences between model-free learning, its neural substrates, and moral judgments. Theoretical work has proposed links between model-based/model-free learning and moral judgment (3, 28, 29, 83), but empirical support for such links has been scarce. We probed two aspects of moral judgment. First, we examined individual differences in the endorsement of instrumentally harming one person to save many others—a key component of utilitarian ethical theories. Consistent with our predictions, as well as work highlighting a link between dlPFC activity and utilitarian judgments (76), we found a positive relationship between endorsement of instrumental harm and dlPFC–sgACC connectivity when switching choices after causing harm to others. We also observed that those who most strongly rejected instrumental harm were the most model-free when learning to avoid harming others.

Second, we used a task that asked participants to judge how morally wrong it would be to perform a series of violent actions that varied independently in terms of how much suffering they would inflict (“outcome sensitivity”) vs. how aversive they felt to perform (“action sensitivity”) (77). We found that individual variability in both the behavioral and neural signatures of model-free learning were specifically correlated with outcome sensitivity, but not action sensitivity. Those people whose moral wrongness judgments were more sensitive to the severity of harmful outcomes were less likely to repeat decisions that harmed others and showed stronger model-free prediction error signals in the thalamus and caudate and stronger responses in sgACC when repeating decisions that previously avoided harming others. Model-free learning has previously been suggested to explain why actions that typically harm others feel aversive to perform, even when they are not actually harmful (3, 28). Thus, one might expect that a greater tendency to engage in model-free moral learning should predict action sensitivity in moral judgments, rather than outcome sensitivity. However, model-free learning is directly sensitive to recent outcomes (18) and in the context of our task manifested as a tendency for choices to be immediately sensitive to harmful outcomes for others. Thus, individual differences in sensitivity to others’ harm could be commonly associated with model-free moral learning and outcome sensitivity in moral judgments. Overall, these findings provide exploratory evidence linking model-free learning to individual differences in moral judgments, effects that should be tested more extensively in larger samples. In particular, it would be worthwhile to probe how model-free learning relates to utilitarian and deontological moral judgments using measures that can dissociate the two (84–86).

More broadly, our findings highlight differences in the neurocognitive mechanisms engaged in learning to avoid harming oneself vs. others that underscore the unique demands of social decision making. One important feature of decisions that affect others (as opposed to oneself) is that it is far more difficult to build a model that incorporates others’ preferences and beliefs than a model that captures only one’s own preferences. Furthermore, it is impossible to evaluate the accuracy of such models, given that the subjective experiences of others are fundamentally unknowable (87, 88). Utilitarian approaches to moral decision making that involve maximizing well-being for all sentient beings (89) may thus be computationally intractable for the model-based system. Rule-based approaches, like those enshrined in deontological theories of morality (90), circumvent the need for complex model building and may be socially adaptive even for simple social decisions like the ones studied here. Regardless of whether such a strategy is adaptive, it remains an open question whether it is normatively appropriate. In addition, whether the prioritization of model-free learning extends to other kinds of social decisions, such as acting to obtain rewards for others, avoiding monetary losses, or, indeed, even social decision making in nonhuman species, is an important topic for future study.

Finally, there are some limitations to our study that should be acknowledged. We specifically designed our study such that the decider participants never met or had any information about the receiver, in order to control for potential motivations of reputation and reciprocity on the decider’s learning and decision making. While this allowed us greater control over participants’ motivation for avoiding harming others, it does not allow us to examine how social knowledge of others influences moral learning. Because it may be more straightforward to build a model that incorporates others’ preferences in situations where those preferences are highly familiar, it could be that people become more model-based for others in such situations, such as choosing on behalf of a romantic partner or sibling, which could be tested in future studies.

In order to compare the relative balance between model-based and model-free strategies when learning for oneself vs. others, it was necessary to use a variant of the two-step task that matched success rates for the two strategies (see *SI Appendix*, *Experimental Note in Introduction*). However, a limitation of this variant of the task is that a purely model-based learner could learn, over the course of the task, that engaging in a model-based strategy is not worth the effort, and thus come to masquerade model-free behavior over time. This is unlikely to be a general problem here, as we did not observe increases in model-free behavior over time at the group level, nor was there a relationship between model-basedness and the degree to which model-free or model-based behaviors changed over time. Future studies should investigate whether the findings reported here generalize across different kinds of learning settings that impose different costs and benefits on model-based and model-free learning. Moreover, while the two-step task we employed allows the assessment of model-free and model-based learning strategies, a hybrid strategy of using both model-free and model based strategies is the most common behavior demonstrated on the task (e.g., refs. 20 and 32) and also what we observed here.

Overall, we observed that when learning to avoid harm to others (vs. self), participants showed a stronger relative balance toward model-free over model-based learning. Multiple model-free learning signatures were apparent in behavior as well as cortical and subcortical areas that distinctly process harm avoidance for others compared to self. These findings could have important implications for theories of learning and moral decision making as well as disorders associated with impaired avoidance learning and social cognition.

## Methods

### Participants.

All participants gave written informed consent and the study was approved by the University of Oxford Medical Sciences Division Ethics Committee. Forty-one right-handed, healthy adults were recruited through university participant databases. Exclusion criteria included previous or current neurological or psychiatric disorder, nonnormal or noncorrected-to-normal vision, previous participation in studies involving social interactions and/or electric shocks, and contraindications that prohibited MRI scanning. Participants were compensated at a rate of £15 per hour. All participants played the role of the decider. One participant was excluded as they reported that they did not believe their decisions would affect another person in the postscanning debrief. Three participants were excluded because the logistic regression analysis of their choice behavior did not converge. In two of these participants, this was because they had less than 5% switch trials; in the third participant, there was a very high correlation (>0.8) between the outcome and transition (common vs. rare) regressors on switch trials. One participant was excluded from the fMRI analysis due to distortions in the scan caused by metal artifacts (braces) and another for excessive head motion (movement of greater than 1 mm in any direction in more than 10% of the total time series). This left a final sample of 36 participants for behavioral analyses (16 female, 20 male, age 18 to 36 y) and 34 participants (16 female, 18 male, age 18 to 36 y) for the parametric fMRI analyses. For the stay/switch analysis, one further participant was excluded for having no variance in at least one regressor, making their fMRI GLM inestimable. With 33 subjects we had 80% power to detect a “medium” effect size of *d* = 0.50 at alpha = 0.05 (two-tailed), an effect size smaller than typically reported in this field, indicating sufficient power.

### Procedure.

Participants attended a 3.5-h experimental session after undergoing substantial prescreening. We administered an extensive pain thresholding procedure which was based on previous studies of self and other pain processing (4, 5). The pain thresholding procedure allowed us to control for heterogeneity of skin resistance between participants to ensure the delivered shocks would be rated at a matched subjective level of pain intensity and also to provide participants with full experience of the shocks before the learning task to ensure their choices were truly guided by knowledge of the pain and no-pain outcomes. Participants were then assigned to roles of either “decider” or “receiver” using a role assignment procedure that has been used in several previous studies (see refs. 5 and 78). Briefly, participants were instructed to wear colored rubber gloves to hide their identity. They then stood either side of a door and waved to one another so that they knew another person was there but could not discern any information about the other participant’s age or gender. Next, a coin was flipped to decide who would draw a ball out of a box first and then each participant drew a ball. The experimental participant was then told that the color of their ball meant they had been assigned to the role of decider. Before completing the task in the scanner participants performed a practice task of one block that did not specify whether outcomes were for self or other and they were told no actual shocks would be received during the practice. This practice task, which used stimuli not used in the experimental task, allowed participants to become familiar with the transition structure of the task, that they were told would remain the same in the main experiment. The main experiment consisted of four blocks of 68 trials (136 trials for self and 136 trials for other) and lasted ∼45 min. This resulted in an analysis of a total of 9,792 choices across the 36 participants in our experiment.

### Experimental Task.

We adapted features from two variants of a task designed to distinguish model-free and model-based learning (20, 27) (Fig. 1 and *SI Appendix*, *Experimental Note in Introduction*). Participants were presented with two fractal images that probabilistically led them to one of two “states” where they were required to make a second choice that was followed by a symbol indicating the receipt of pain or the receipt of no pain (neutral). At the beginning of each block and on each trial, they were told whether they were playing for themselves (“You”), meaning the painful outcomes would be delivered to themselves, or for the other participant (“Receiver”), indicating that the painful outcomes would be delivered to the other participant. In order to match the self and other conditions in terms of pain stimulation, no electric shocks were delivered during the scan. However, participants were told that 10% of the electric shocks that they acquired during the task would be given to themselves at the end of the session and 10% of the electric shocks they accumulated for the other participant would be delivered to the other participant at the end of the scanning session. In order to account for potential differences in pain perception, participants were instructed that for “You” trials we would use the voltage setting that corresponded to their level-8 rating, and for the “Receiver” trials we would use the voltage setting that corresponded to the Receiver’s level-8 rating (full instructions can be downloaded at OSF: https://osf.io/3stp9/files/). At the end of the scan, participants also rated how they felt when obtaining a shock for themselves and the receiver during that task on a 0 to 10 point scale from “very negative” (0) to “very positive” (10). Participants perceived the shocks to be aversive for both self (mean = 3.38, SD = 1.44) and other (mean = 3.30, SD = 1.61) [*t*(36) = 0.279, *P* = 0.782], suggesting that the perception of harm was equivalent. The order with which participants completed the self and other blocks was counterbalanced across participants, as were the experimental stimuli that belonged to self and other blocks, to control for possible confounds in liking of different stimuli. Participants were instructed that the probability of reaching each of the two “states” would remain fixed throughout the task but the probability that each stimulus would deliver pain or no pain would change throughout the task so that they needed to keep on learning. See *SI Appendix*, *Supplementary Text* for further details.

### Statistical Analysis of Behavioral Data.

Analyses of behavioral data were performed in R (version 1.1.423, for linear mixed-effects modeling, lme4, version 1.1-21) and MATLAB 2015b (for logistic regression analyses on the probability of stay and for computational modeling analyses employing hierarchical fitting written with custom MATLAB code; see *SI Appendix*, *Supplementary Text* for further details). For nonsignificant tests, to provide evidence for the null, Bayesian statistics was performed in JASP (https://jasp-stats.org) (91, 92). Default priors from the JASP program were used. The strength of null effects was interpreted using the language suggested by Jeffreys (93). All tests were two-tailed.

### Moral Judgment Measures.

Participants completed the Oxford Utilitarianism Scale (74) and the Harmful Action Outcome scale (77) via an online link in the preceding weeks before the scanning session. See *SI Appendix*, *Supplementary Text* for further details.

### Computational Modeling of Behavioral Data.

For modeling of choice behavior using trial-by-trial updates, we evaluated a number of plausible models fitted either to self and other blocks separately or across self and other blocks combined. Models were fitted using a hierarchical Bayesian model fitting approach described in detail in refs. 94 and 95. It finds the maximum a posteriori estimate of each parameter for each subject using a prior distribution for each parameter which helps to regularize and constrain parameters. The algorithm uses expectation–maximization (96) and parameters were transformed to a logistic or exponential distribution to enforce constraints and ensure normality such that 0<{α,ω}<1, {β,λ}>0.

For formal model comparison, we report the Bayesian information criterion (BIC) based on the log-likelihood and computed the model evidence by integrating out the free parameters [BICint (94, 95); *SI Appendix*, Tables S1 and S2]. Exceedance probabilities were calculated by feeding the BICint into SPM’s function spm_BMS (https://www.fil.ion.ucl.ac.uk/spm/software/spm8/). The winning model was a six-parameter model with αPain, αNoPain, β, and ρ shared but ω split into ωSelf and ωOther. See *SI Appendix*, *Supplementary Text* for further details.

### fMRI Acquisition and Analysis.

Multiband T2*-weighted echo-planar imaging (EPI) volumes with blood oxygenation level–dependent (BOLD) contrast were acquired using a Siemens Prisma 3T MRI scanner. The EPI volumes were acquired in an ascending manner, at an oblique angle (∼30°) to the AC–PC line to decrease the impact of susceptibility artifacts in the orbitofrontal cortex. We used the following parameters. Voxel size 2 × 2 × 2, echo time = 30 ms; repetition time = 1,570 ms; flip angle = 90°; field of view = 216 mm. The structural scan was acquired using a magnetization prepared rapid gradient echo sequence with 192 slices; slice thickness = 1 mm; repetition time = 1,900 ms; echo time = 3.97 ms; field of view = 192 mm × 192 mm; voxel size = 1 × 1 × 1-mm resolution. fMRI data were analyzed using SPM12 (https://www.fil.ion.ucl.ac.uk/spm/) using a standard preprocessing pipeline (see *SI Appendix*, Figs. S7 and S8 and *Supplementary Text* for details and setup of GLMs 1 through 5).

Contrast images from the first level were input into flexible-factorial designs. Following standard procedures, main effects are reported at *P* < 0.05, FWE cluster-corrected across the whole brain after initial thresholding at *P* < 0.001 or *P* < 0.05 FWE-SVC after initial thresholding at *P* < 0.001 for regions where we had a strong a priori hypothesis (66, 67). These areas were defined anatomically and included subcortical regions of the caudate and thalamus (taken from the WFU PickAtlas Toolbox), bilateral posterior TPJ (from ref. 97), the sgACC (areas s24 and 25 from ref. 98), and the dlPFC (areas 46v and 9 taken from ref. 99). These ROIs were also used to confirm anatomical labeling. We also performed a meta-analysis in Neurosynth for the term “pain” that included studies of both observed and experienced pain. This meta-analysis robustly showed activation in the thalamus that overlapped both with the cluster identified in the present study and with the findings of Crockett et al. (4) showing that caudate and thalamus activity reflect the modulation of action values by moral context (*SI Appendix*, Table S8). We therefore also used this functional response in the thalamus as an additional functional ROI to corroborate our anatomical thalamus ROI that showed responses to Other PE > Self PE. We also applied a false discovery rate correction (FDR) for the number of ROI corrections. All ROI comparisons remained significant (*P* < 0.05) when controlling for the number of comparisons using FDR.

## Supplementary Material

Supplementary File

## Data Availability

All anonymized behavioral data and code used to generate the figures can be downloaded at OSF (https://osf.io/3stp9/files/). All code used to run the computational modeling can be downloaded at OSF (https://osf.io/3stp9/files/). Unthresholded statistical maps can be downloaded at NeuroVault (https://identifiers.org/neurovault.collection:8797).
