# Supplementary material for: Model-free decision making is prioritized when learning to avoid harming others
Source: Proc Natl Acad Sci U S A. 2020 Oct 14;117(44):27719–30. doi: 10.1073/pnas.2010890117 (PMC7959560; doi:10.1073/pnas.2010890117)
Supplement: Supplementary File [file pnas.2010890117.sapp.pdf]

PNAS
www.pnas.org

Supplementary Information for “Model-free decision making is prioritised when learning to avoid harming others”

Patricia L. Lockwood, Miriam Klein-Flügge, Ayat Abdurahman, & Molly J. Crockett

Correspondence should be addressed to:
Patricia L. Lockwood, p.l.lockwood@bham.ac.uk
Miriam C. Klein- Flügge, miriam.klein-flugge@psy.ox.ac.uk
Molly J. Crockett, molly.crockett@yale.edu

This PDF file includes:

Supplementary text
Figures S1 to S8
Tables S1 to S6
SI References

Supplementary Text

Experimental note in introduction

When designing our study, we considered multiple variants of two-step tasks that have previously been used in research[1–4]. Our aim here was to specifically design our task to assess people's 'relative balance' between engaging in model-free and model-based strategies using a paradigm that yields equal frequencies of pain versus no pain outcomes independent of the level of model-basedness and thus did not 'push' people towards being more model based. We ultimately opted for a hybrid version of the original Daw et al[4] paradigm and the more recently suggested version by Kool et al[2], including some of the benefits of both of these variants of the task.

We chose to adopt one important feature from the original paradigm by Daw et al.[4] and designed the current paradigm such that there was no incentive to be model-based. In other words, model-free and model-based learning strategies yielded overall similar proportions of positive versus negative outcomes. This meant that participants could not avoid more pain by adopting a model-based over a model-free strategy. This was an important feature for two reasons. First, there is extensive evidence that people value others' outcomes differently from their own[5,6] and are willing to exert more effort to benefit themselves than others[7] If we had adopted a more recent variant of this task by Kool et al. where the model-based strategy more effectively obtained desired outcomes (e.g.,[2]) and observed differences in model-based learning for self vs. other, we would have been unable to rule out the possibility that such differences were due to differential utilities in outcomes for self vs. other. Second, this aspect of the original variant of the task is optimised to measure people's 'relative balance' or 'natural tendency' between engaging in model-free vs. model-based learning. In their paper describing the newer version of the two-step task, Kool et al themselves state that "even though there is no significant relationship between reward and model-based control [in the two-step task], this does not undermine the usefulness of the task for measuring the relative balance of model-based and model-free strategies (page 6-7)." The authors therefore highlighted the usefulness of using a variant more closely based on the original Daw paradigm to assess the relative balance between systems which was the main aim of the current study. By contrast, the newer version[2] focusses on the trade-off between cognitive demand and 'accuracy' (here successfully avoiding harm), which was not the focus of the current study.

However, we also adopted some of the improvements in task design suggested by Kool et al[2]., including changes in drift rate and drifts that include more extreme probabilities to facilitate learning. Specifically, we made the drifting of the random walks faster (0.2 as in Kool, rather than 0.025 in Daw) and bounded the reward(pain) rate between 0-1 rather than 0.25-0.75 as suggested by Kool et al. The drift rate referred to the standard deviation of the normal distribution from which the random noise that was added at each timepoint was drawn when generating the random walks. The first modification allowed us to assess learning within a smaller number of trials (138 per agent) than in Daw et al[42] (201 trials) but comparable to Kool et al[2] (125 trials). The second modification allowed us to do this whilst ensuring the task was not too difficult. Also, because there are currently no studies involving neural data for the Kool et al paradigm we wanted a task that could be used, in part, to replicate the neural findings described in Daw et al.[4].

Supplementary Results

Linear mixed effect model of behavioural data

We validated our behavioural results by repeating our analysis using linear-mixed effects models with the lme4 package in R, which ensured we had good estimates of random effects and accounted for variability in behaviour using Bound Optimization by Quadratic Approximation (see Methods). We found all results remained the same (glmer(Switch_Stay~NoPain_Pain*Transition*Recipient+(1+NoPain_Pain*Transition*Recipient I Subject)), (main effect of outcome (model-free) Z=4.154, *p*<.001) ; Outcome x transition interaction (model-based) Z=3.841, p=.001; outcome x recipient Z=-2.048, p=0.041; [outcome x transition (model-based)] x recipient Z=0.255, p=0.80).

Bayesian 1st level analysis comparing prediction error and outcome only models

We ran a Bayesian 1st level analysis in SPM12 to compare exceedance probabilities for the PE model (Model 1) and a model based on outcome only (Model 2, no pain/pain). We constructed GLMs that were identical except for whether the onset of each outcome had a parametric regressor modelling model-free prediction errors or outcomes (1 or 0). We note that PE and outcome regressors were highly correlated (r^2=.83). We then compared the exceedance probabilities at the group level for each of the peak coordinates in thalamus and ventral striatum reported in the manuscript. This analysis showed that one peak in the thalamus was best explained by a PE model (M1, xp=0.97), one by the outcome model (M2, xp=1) and another equally by both models (xp=0.43 and xp=0.57). In the striatum, one peak (right) was best explained by a PE model (Table S2) whereas another (left) was best explained by an outcome model (Table S2). Therefore, the results suggest anatomical specificity in tracking PEs and outcomes. Both variables were encoded in thalamus and striatum, but in anatomically distinct locations.

TPJ BOLD and model-free and model-based behaviour

In GLM2, we observed the inverse pattern to the one seen in sgACC in right TPJ (x=54, y=-38, z=34, Z=3.56, K=39, *p*=.03, FWE-SVC, Fig. S2). In other words, the signal in TPJ was opposite to what would be predicted by model-free influence. This region was more active during switch relative to stay choices on the current trial, following a 'no pain' outcome on the previous trial, in the 'other' condition specifically. However, in GLM3 where we tested whether there was a significant outcome x transition interaction (model-based), as well as a main effect of outcome (model-free), we found no suprathreshold voxels in the independent anatomical TPJ ROI.

Switch/stay additional analyses

In the switch-stay analysis we identified sgACC and TPJ activity after receiving no pain for other (Figure 5 and Fig. S2.). There were no significant results at the whole brain level or in any of our ROIs after no pain for self, or pain for either agent. Due to the coding of switch-stay as a parametric modulator we were unable to statistically assess a full 2 (self/other) x 2 (switch/stay) x 2 (no pain/pain) interaction. However, in sgACC, we additionally confirmed using post-hoc t-tests that there was indeed no response for these three parametric modulators (self no pain ($t(32)$=-.56, *p*=.58, BF_{01} = 4.6 [substantial in support of null]), self pain ($t(32)$=1.52, *p*=.14, BF_{01} = 1.89 [anecdotal in support of null]), other pain ($t(32)$=-.05, *p*=.96, BF_{01} = 5.37 [substantial in support of null])). This was also true for right TPJ: it responded to stay more than switch after no pain for other but there was no significant effects in any of the other three conditions (self no pain ($t(32)$=.95, *p*=.35, BF_{01} = 3.56 [substantial in support of null])), self pain ($t(32)$=1.09, *p*=.29, BF_{01} = 3.12 [substantial in support of null])), other pain ($t(32)$=-.065, *p*=.95, BF_{01} = 5.36 [substantial in support of null])).

Testing for differences in neural responses to self and other at decision time

We ran an exploratory GLM to test whether participants showed any difference in overall response to self vs other at decision time, perhaps consistent with such processing requiring a greater cognitive load. We modelled the onset of self and other choices separately and ran simple contrast to directly compare them. No brain areas survived whole brain correction or showed a significant response in any of our ROIs.

Model-free influence at choice additional analyses

Although the results related to Figure 5 in the main text are consistent with a model-free influence in sgACC, a decisive test would be to also show a BOLD effect of outcome, but no significant outcome x transition interaction, analogous to the behavioural analysis. Therefore, in an additional GLM (GLM3) we instead modelled onsets separately for stay versus switch choices with two parametric regressors each – a model-free (outcome, (no pain/pain)) and a model-based (outcome x transition (no pain/pain x common/rare transition)) regressor. We allowed the model-free and model-based regressors to directly compete for variance (correlations were all <0.4). We found that sgACC activity for other reflected the model-free effect, responding most strongly to switch choices following pain relative to no pain, in a cluster that overlapped with our analysis in GLM2 (x=0, y=36, z=6, K=29; Z = 3.46, *p*=.024, FWE-SVC for an anatomical sgACC ROI after initial thresholding at *p*<.001). Importantly, within this independent anatomical ROI, there was no significant parametric modulation that reflected the outcome x transition interaction (*p*=.12, $t(32)$ =1.59, BF_{01}=1.7, *d*=0.28), and the model-free and model-based estimates were also

significantly different from one another (p=.017, t(32) = -2.5, d = -.44). We note that it would be ideal to conduct an analysis on just the rare trials to provide further support that this signal reflects a model-free influence. However, by design, there are few rare trials, and such an analysis would not be well powered.

Psychophysiological Interaction analyses

For completeness we also ran an additional set of GLMs that used seed regions in brain areas apart from sgACC that showed specificity of model-free processing for other compared to self (Thalamus and TPJ). We defined a seed region in these two areas using a 6mm sphere based on the peak co-ordinates from our analyses. We then extracted the physiological variable and the psychophysiological interaction terms for other prediction error > self prediction error (peak in thalamus/caudate) and stay vs. switch after no pain for other. These PPI terms were entered into the GLMs along with all previous regressors that specified the events of our study. In all PPI GLMs, six head motion parameters modelled the residual effects of head motion as covariates of no interest. Neither area showed significant connectivity with any part of the brain at the whole brain or small-volume corrected level.

Profile of dlPFC response

We ran two additional GLMs (GLM 4.1 and GLM 4.2) to assess whether dlPFC responses were specific to connectivity with sgACC or also showed a main effect. In the first we modelled the onset of stay vs. switch trials separately for the self and other condition to test whether dlPFC was more active for switch vs. stay trials overall. We confirmed that the model was estimable (correlations between regressors: all r's<l.032l). This analysis showed no significant voxels at the whole brain level or in any of our ROIs. In the second GLM, we coded each participants' choice as consistent versus not consistent with model-free behaviour (1=stay after no pain, switch after pain; -1=stay after pain, switch after no pain) and again observed no significant voxels at the whole brain level or in any of our ROIs. These findings suggest that dlPFC effects were related to differential connectivity with sgACC, rather than reflecting a main effect of switch/stay during the task or a main effect of model-free processing regardless of connectivity with sgACC.

Correlations between shock aversiveness and ω parameter

We also tested whether ratings of the aversiveness of shocks for self and other, or the difference between self and other, correlated with the model-free x recipient interaction and with the ω parameters for self and other. No correlations were statistically significant (all r's <.09, all ps > .61). Instead, the strongest correlation with the model-free by recipient interaction was with the sensitivity of moral judgments to harmful outcomes, consistent with the idea that aversion to harming others drove the model-free effects rather than the perception of the aversiveness of shocks.

Supplementary discussion

Time pressure, reaction times and social decision-making

Previous studies have suggested that time pressure can alter social decision-making such that increased time pressure can make people more cooperative[8,9]. Although in our experiment average RTs (1.2 seconds) were well below the allotted time available to make decisions (2.5 seconds), it would be interesting to compare a self-paced version of the task with one where participants are under time-pressure to examine whether more time to deliberate would reduce participants' model-free behaviour for others.

Previous research using the two-step task in combination with eye tracking has suggested that subjects who are "model-free" deliberate more at the time of choice whereas those that are "model-based" may make their choice about which first-stage cue to select prior to its onset[10]. Whilst we did not observe sgACC or dlPFC activity when we modelled switch-stay decisions at the time of the outcome instead of the choice, or any differences in reaction times between conditions, it would be interesting to examine whether an aversion to harming others is reflected in eye gaze deliberation.

Alternative accounts of increased model-free decision making for others

An potential alternative explanation for our behavioral finding showing that participants were more model-free for other than self is that model-based learning is effortful[2], and people might choose to put in less effort to benefit others[7]. As described in the main text, this explanation seems unlikely because being more model-free overall should equally influence trials that lead to pain or no pain. However, in our study, model-free moral learning was specifically driven by a lower probability of repeating choices that harmed others (pain outcome), while there was no difference between self and other on trials that avoided harm (no pain outcome). Furthermore, we did not observe differences in choice consistency (captured by the temperature parameter in our model) during learning for self vs. other. Since choice consistency is related to task engagement, if our behavioral effects reflected reduced effort for others, we might expect to see lower choice consistency when learning for others than self. However, we acknowledge that the link between choice consistency and cognitive control is not completely straightforward[11]. RT analyses also showed that choices were not slower for other compared to self trials, which again might be expected if effort was different between self and other.

Opposing Prediction error signals in the thalamus

Of note, the thalamus tracked prediction errors for self and other in opposing directions, signalling a positive PE when avoiding pain for others, but signalling a negative PE when causing harm to oneself. Whilst we interpret these results with caution to avoid problems of reverse inference, one possible explanation is that participants were differentially invoking optimism/pessimism biases for self and other. If participants were pessimistic about avoiding harm to others but optimistic about avoiding harm to themselves, this may have been reflected in opposing encoding in the thalamus. It would be interesting for future studies to employ designs used to study optimism and pessimism biases and examine whether these might be able to explain learning to avoid harm for self and other.

Overlap between behavioural and neuroimaging results

Of note, our main neural result at decision time in sgACC was specific to no pain trials, whereas behaviourally we observed differences between self and other more strongly on pain trials, as evidenced by increased switching after pain caused to others. However, overall there was consistency between our behavioural and neural effects. First, our neural finding of distinct model-free prediction error signalling for self vs. other outcomes in the ventral striatum and thalamus/caudate is in line with our behavioural effect of more model-free behaviour for others, as the prediction error tracks responses parametrically to no pain vs. pain outcomes. Second, our behavioural effect (more model-free for other than self) does correlate with responses in sgACC, with those people who are more model-free for other having stronger sgACC responses to stay vs. switch after no pain for other.

Finally, we ran an additional analysis to further understand our results in sgACC (see SI Text Model-free influence at choice additional analyses for full statistical details). In parallel to our behavioral analysis, we coded a GLM where we modelled parametric regressors of the choice at the current trial as a function of (a) outcome on the previous trial (pain/no-pain, the model-free influence) and (b) an interaction between outcome (pain/nopain) and transition (rare/common) on the previous trial (the model-based influence). We then looked at these two parametric regressors separately for trials when participants switch or stay, or by pooling across switch/stay decisions. We used an anatomical ROI in sgACC that was not biased in any way towards finding model-free or model-based effects.

Our results demonstrate effects in sgACC consistent with a model-free, but not a model-based influence on choice, in a cluster overlapping with our original analysis reported in the manuscript. In other words, sgACC activity tracks more strongly with decisions to switch than decisions to stay following pain than no pain for other (model-free effect: contrast of pain outcome (coded as -1) vs. no pain outcome (coded as 1), regardless of transition type). We note that this is highly consistent with our behavioural effect, whereby participants are more likely to switch following pain than no pain to others, regardless of transition type.

By contrast, the model-based regressor that codes the outcome x transition interaction shows no significant parametric modulation in sgACC, with these two effects in sgACC significantly different from one another. Taken together, these results suggest consistency between our behavioural and neuroimaging analyses (See Fig S3. for additional details).

Supplementary Methods

Detailed description of full 7-parameter model

We refer to Daw et al.[4] for a more detailed description of the learning model but repeat a short description with the formulas here for completeness.

We denote trials with *t*, states with *i* (A=1st stage, B/C=2nd stage), actions as *j* and rewards as *r*. Model-free updating was described using simple TD learning, with the only free parameters α denoting the stage 1 and 2 learning rates:

$$Q_{TD}(s_{i,t}, a_{i,t}) = Q_{TD}(s_{i,t}, a_{i,t}) + \alpha_i \delta_{i,t}$$

where the prediction error was defined as

$$\delta_{i,t} = r_{i,t} + Q_{TD}(s_{i+1,t}, a_{i+1,t}) - Q_{TD}(s_{i,t}, a_{i,t})$$

The effect of the eligibility parameter λ was to update first-stage actions by second-stage prediction errors

$$Q_{TD}(s_{1,t}, a_{1,t}) = Q_{TD}(s_{1,t}, a_{1,t}) + \alpha_1 \lambda \delta_{2,t}$$

Model-based learning was the same as model-free learning at the second stage. At the first stage, model-based values were modelled as

$$Q_{MB}(s_A, a_j) = P(s_B|s_{A,}a_j) \max_{a \in \{a_A, a_B\}} Q_{TD}(s_B, a) + P(s_C|s_{A,}a_j) \max_{a \in \{a_A, a_B\}} Q_{TD}(s_C, a)$$

where $P(s_B|s_{A,}a_A)$ and $P(s_C|s_{A,}a_B)$= 0.7 and $P(s_C|s_{A,}a_A) = 1 - P(s_B|s_{A,}a_A)$ and $P(s_B|s_{A,}a_B) = 1 - P(s_C|s_{A,}a_B)$.

Model-based and model-free action values were arbitrated using the free weighting parameter ω:

$$Q_{net}(s_A, a_j) = \omega Q_{MB}(s_A, a_j) + (1 - \omega) Q_{TD}(s_A, a_j)$$

Finally, the probability of choice was computed using a softmax function with inverse temperature parameters β for both stage 1 and 2, and perseverance parameter ρ.

$$P(a_{i,t} = a|s_{i,t}) = \frac{\exp\left(\beta_i\left[Q_{net}(s_{i,t}, a) + \rho \, \text{rep}(a)\right]\right)}{\sum a'\left(\exp\left(\beta_i\left[Q_{net}(s_{i,t}, a') + \rho \, \text{rep}(a')\right]\right)\right)}$$

Here, rep(a)=1 if the 1st stage choice was the same as one the previous trial, and 0 otherwise.

Computational modelling of behavioural data

For modelling of choice behaviour using trial-by-trial updates we proceeded in two steps. First we fitted a range of plausible models separately to self and other blocks. This was to probe whether the same model would win for self and other blocks, allowing us to rule out participants might employ entirely different strategies in the two block types. The following models were fitted:

<u>(1) 7-parameter: full model specified by Daw et al. using parameters:</u> learning rates for stage 1 and 2 (αS1, αS2), temperature parameters for stage 1 and 2 (βS1,βS2), a perseverance parameter (ρ), an eligibility trace (λ) and a model-free/based weighting parameter (ω); for full details of model see Supplementary information and[4]

(2) 6-parameter model, as (1) but with λ=1 (λ was shown to have a high mean value and small variance in previous work e.g.[12])
(3) 5-parameter model, as (1) but with only one α and β for stage 1 and 2
(4) 4-parameter model, as (3) but with λ=1
(5) 5-parameter model, as (4) but with two learning rates for pain and no pain outcomes (αPain, αNoPain)

Models were fitted using a hierarchical Bayesian model fitting approach described in detail in[13,14]. It finds the maximum *a posteriori* estimate of each parameter for each subject using a prior distribution for each parameter which helps to regularise and constrain parameters. The algorithm uses Expectation-Maximization (EM)[15] and parameters were transformed to a logistic or exponential distribution to enforce constraints and ensure normality such that $0<\{\alpha,\omega\}<1$, $\{\beta,\lambda\}>0$.

For formal model comparison, we report the Bayesian Information Criterion (BIC) based on the log-likelihood, and computed the model evidence by integrating out the free parameters (BICint[13,14]; Tables S3 and S4). Exceedance probabilities were calculated by feeding the BICint into SPM's function spm_BMS (http://www.fil.ion.ucl.ac.uk/spm/software/spm8).

The five-parameter model with separate learning rates for pain and no-pain outcomes best explained behaviour in both self and other conditions (Table S3). We report the difference between the best-fitting parameters, but this method has a caveat. Because of the nature of hierarchical fitting, which uses separate priors for self and other parameters, this method is somewhat biased towards finding differences. Meanwhile, fitting self and other parameters using the same priors, is overly conservative and biased against finding differences.

To resolve whether the parameter ω differed between self and other blocks, in line with results from the basic logistic regression analyses, and without introducing any such biases, in a second step, we therefore fitted three models to the merged data of both self and other blocks:

1. 5-parameter model (as (5) above) with all parameters shared between self/other
2. 6-parameter model with αPain, αNoPain, β and ρ shared but ω split into ωSelf and ωOther
3. 7-parameter model with αPain, αNoPain, β shared and ρ and ω split into ρSelf, ρOther, ωSelf and ωOther

As described above, model comparison was performed based on BICint values. The mean parameter estimates are shown in Table S4. We also simulated data from our participant schedules and showed that we had reliable parameter recovery[16,17].

Parameter recovery

Because schedules had been optimized for the seven-parameter model, but our winning model involved five parameters, with two separate learning rates for pain and no pain outcomes, one inverse temperature parameter and a fixed λ=1, we tested whether we could recover parameters for this model from simulated data for which we knew the ground truth. For the five parameters (αPain, αNoPain, β, ρ, ω), we simulated behaviour using the same schedule given to our participants. We used a wide range of parameter values (total of 4 x 4 x 3 x 3 x 4 = 576 combinations), from a grid of values in the ranges: α=[0.2 0.4 0.6 0.8]; β= [2 3.5 5], ρ=[-0.5 0 0.5] and ω= [0.2 0.4 0.6 0.8]. We added noise to each of the five parameters for each simulated agent (from a standard normal distribution multiplied by 0.1) to improve our coverage of possible parameter values. After having generated the behaviour, we refitted the simulated behaviour using fminunc in Matlab. We used the best fit from 10 random starting configurations to avoid local minima.

The correlations between the true simulated and fitted parameter values were: αPain, αNoPain= 0.88; β=0.82; ρ=0.96; ω= 0.8. Thus, parameter recovery was reliable for all parameters.

Statistical analysis of behavioural data

For Fig 2a, we calculated the % of stay choices after common or rare transitions following pain or no pain outcomes (2x2). For regression analyses using lme4 in R, we coded Stay as 1 and Switch as 0 and created regressors for transition type, outcome, outcome x transition type, agent x outcome and agent x outcome x

transition type. We used Bound Optimization by Quadratic Approximation (bobyqa) with 1e5 function evaluations. We examined bivariate associations between the interactions with agent from the regression analyses and neural responses with individual differences in utilitarianism using the Oxford Utilitarianism Scale (OUS)[18] and action and outcome sensitivity from the Harmful Action Outcome Scale[19].

Moral Judgment Scales

The OUS-IH consists of 4 items reflecting a relative willingness to cause harm to others in order to bring about the greater good (e.g., "It is morally right to harm an innocent person if harming them is a necessary means to helping several other innocent people"). Participants rated these items on a 7-point scale (*1 = strongly disagree, 7 = strongly agree).* A mean score was then computed for all participants.

For the HAO participants were presented with a scenario about two people, Carl and John. They are told John is terminally ill and sincerely wants to die and has asked another person, Carl, to perform a mercy killing. Participants then rated how morally wrong each of the 23 methods of killing were on a scale from 1 – the least morally wrong, to 10 – the most morally wrong. In a previous study[19] participants were asked to rate the action value and outcome value of these different methods of killing. To assess action value, the researchers asked participants to rate how *upsetting* it would be to "act out" performing each behavior as though it were part of a movie script. To assess outcome value, they asked them to rate how much *suffering* each act would impose. We used the mean action and outcome scores derived from this initial paper to predict the 'wrongness' scores in our current sample. This analysis created two different beta weights for each participant corresponding to the action and outcome sensitivity, respectively.

fMRI pre-processing and set-up of GLMs 1-5

Images were realigned and unwarped using a fieldmap and co-registered to the participant's own anatomical image. The anatomical image was processed using a unified segmentation procedure combining segmentation, bias correction, and spatial normalization to the MNI template using the New Segment procedure; the same normalization parameters were then used to normalize the EPI images. Lastly, a Gaussian kernel of 8 mm FWHM (SPM default) was applied to spatially smooth the images.

Before the study, example first-level design matrices were checked to ensure that estimable GLMs could be performed with independence between the parametric regressors: value difference at the first-stage choice, the state prediction error at stage 2, and a model-free prediction error at the time of the outcome (Figure S6). This allowed us to look at value and prediction error responses independent from one another. We also tested a GLM that coded switch vs. stay trials as a parametric modulator at the time of choice dependent on the previous outcome. Again this GLM could be estimated with independence (See Figure S7). We convolved these different event types with SPM's canonical haemodynamic response function. All events were modelled as stick functions with 0 duration.

For GLM1, each of these regressors was associated with parametric modulators taken from the computational model. At the time of the first stage choice this was the value difference from the hybrid model combining model-free and model based learning. At the time of the second stage choice this was the state prediction error for the transition from stage 1 to stage 2, and at the time of the outcome this was the model free prediction error (since the behavioural differences were in the model-free parameters). In all cases, values were modelled separately for the onsets of self and other trials. As in20, we fixed the parameters to the average values for self and other (Table S5 and S6) but allowed ω to vary.

For GLM2 we modelled whether participants stayed or switched at the first-stage choice relative to the outcome on the previous trial, i.e. no pain or pain. Due to the smaller number of trials included in this analysis we coded stay and switch as a parametric regressor with values of 1 assigned to switch and -1 assigned to stay. One participant did not have a trial in at least one of these regressors and was therefore excluded from the stay-switch analysis. For all GLMs in some participants, an extra regressor modelled all missed trials, on which participants did not select one of the first-stage choices.

GLM3 was designed to confirm that the BOLD effects in sgACC were indeed best explained by a model-free, rather than a model-based, influence. This GLM was similar to GLM2 but instead of splitting regressors by

the outcome on the previous trial, we modelled the onsets of switch and stay choices (with respect to the choice on the previous trial), for both self and other. Each of these regressors was assigned two parametric modulators, a parametric regressor coding the outcome of the previous trial irrespective of transition (pain/no pain, model-free) and a second regressor that coded the outcome by transition interaction on the previous trial (outcome x transition, model-based).

GLM4 and GLM5 corresponded to our psychophysiological interaction analyses. We defined a seed region in the sgACC using a 6mm sphere based on the peak co-ordinates from our analyses (for completeness we also ran PPI analyses using seed regions in thalamus and TPJ, see SI Text). We then extracted the physiological variable and the psychophysiological interaction terms for stay vs. switch after no pain for other (GLM4) and stay vs. switch after no pain for self as a control analysis (GLM5). These PPI terms were entered into the GLMs along with all previous regressors that specified the events of our study as described above. Finally GLM 4.1 and GLM 4.2 were used as control analyses for GLM 4. GLM 4.1 modelled the onset switch vs. stay decisions at the time of the outcome rather than the time of decision. GLM 4.2 modelled whether the choice participants made on the current trial was more consistent with the recommendations of model-free or model based behaviour (see SI Text). In all GLMs, six head motion parameters modelled the residual effects of head motion as covariates of no interest. Data were high-pass filtered at 128 s to remove low-frequency drifts, and the statistical model included an AR(1) autoregressive function to account for autocorrelations intrinsic to the fMRI time-series.

Fig. S1.

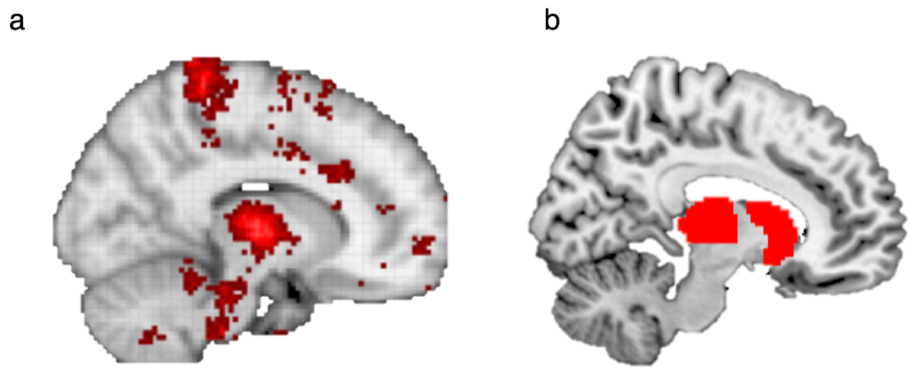

Neurosynth meta-analysis. a) Neurosynth meta-analysis of the term 'Pain' from 516 studies (including both experienced pain and observed pain) showed robust activation in the thalamus that overlapped with the structural thalamus ROI we used to examine specific responses to Self and Other prediction errors. (b) Independent structural small-volume region of interests in the thalamus and caudate.

Fig. S2.

(a) Temporoparietal junction (TPJ) (x=54, y=-38, z=34, Z=3.56, p=.03, FWE-SVC after initial thresholding at p<.001) tracks switch more than stay after no pain for other. (b) Cluster overlaid on an anatomical scan.

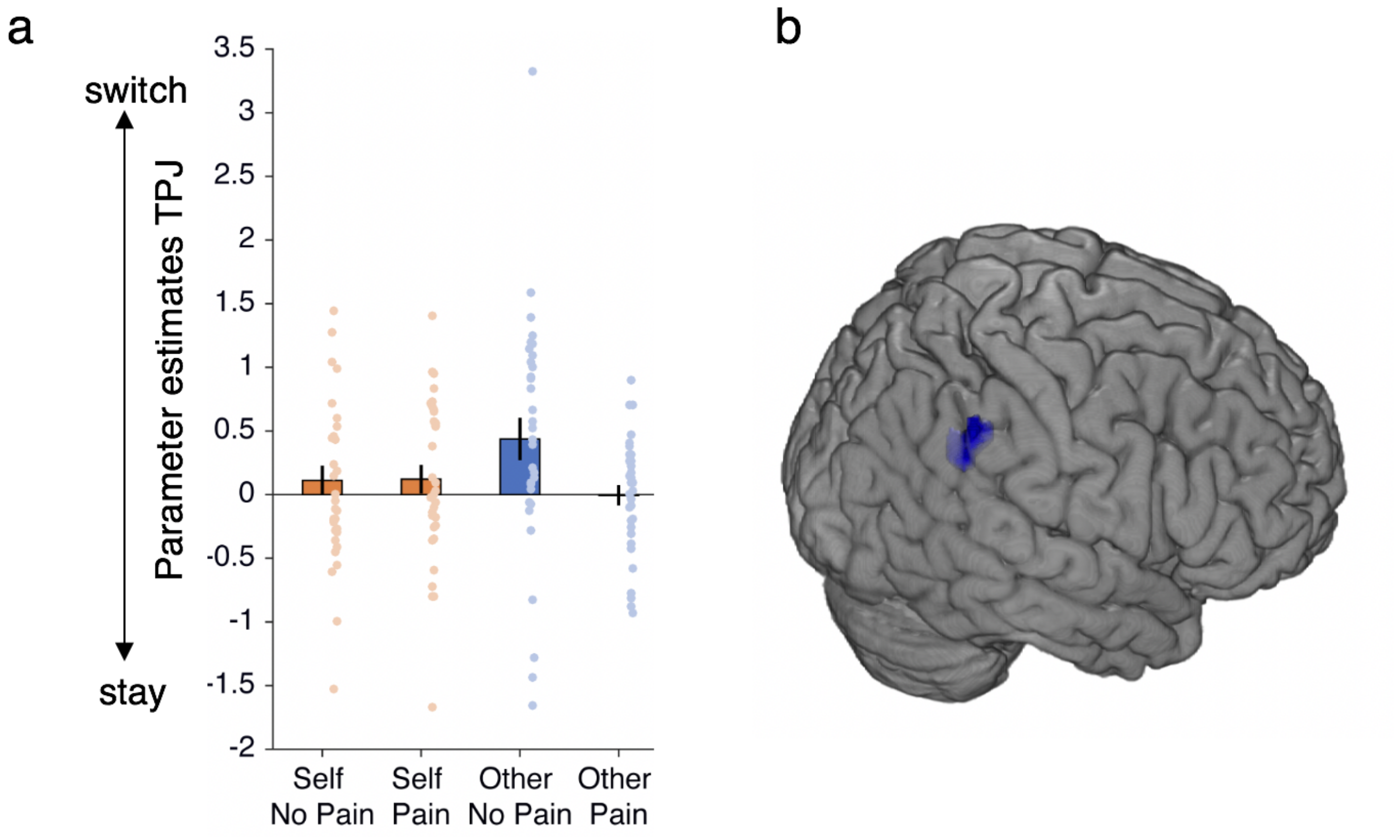

Fig. S3. Hypothesised pattern of model free effects as a function of switch/stay and no pain/pain. In GLM2, we modelled onsets separately for choices following pain versus no pain outcomes, with switch/stay as the parametric modulator, and concluded that sgACC BOLD distinguished between stay versus switch after no pain for others (GLM2, red arrow in the schematic below). In GLM3, we modelled onsets separately for switch and stay choices, with pain/no pain on the previous trial as the parametric modulator, and here we see that sgACC activity distinguishes between switching more after pain compared to no pain to another person (denoted by the yellow arrow in the schematic below). Both of these effects are consistent with a model-free influence on decisions that affect others.

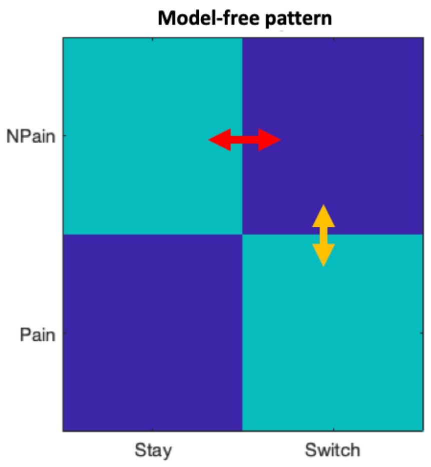

Fig. S4. Bootstrapping analysis for rare trials. The effect of switch versus stay for other after no pain was estimated for rare trials only (blue line) and for 1000 GLMs that were matched, in trial numbers, to the rare trials but relied on a randomly drawn subset of only common trials in each iteration (grey histogram). Rare trials were contributing to the effect of switch vs stay for other after no pain about as much as common trials, supporting our interpretation that sgACC response is consistent with a model-free effect. (parameter estimate from rare trials only: -.042; mean parameter estimate from 1000 models using random subsets of common trials matching the number of rare trials: -.036 +/- SE .002). However, we note that each model in this analysis relied on a small subset of trials and thus, this result should be interpreted with caution.

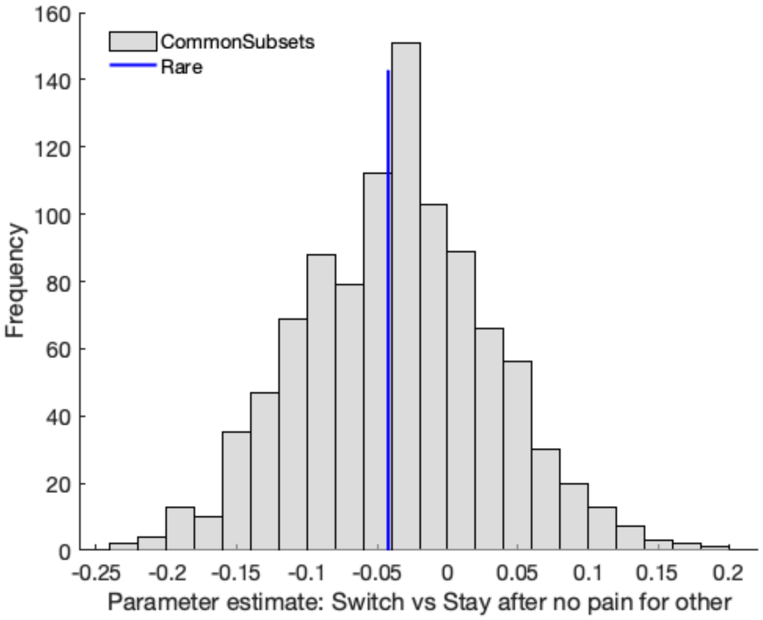

Fig. S5. Correlations between Oxford Utilitarianism Scale (OUS) model-free moral behaviour and dlPFC switch after no pain for other.

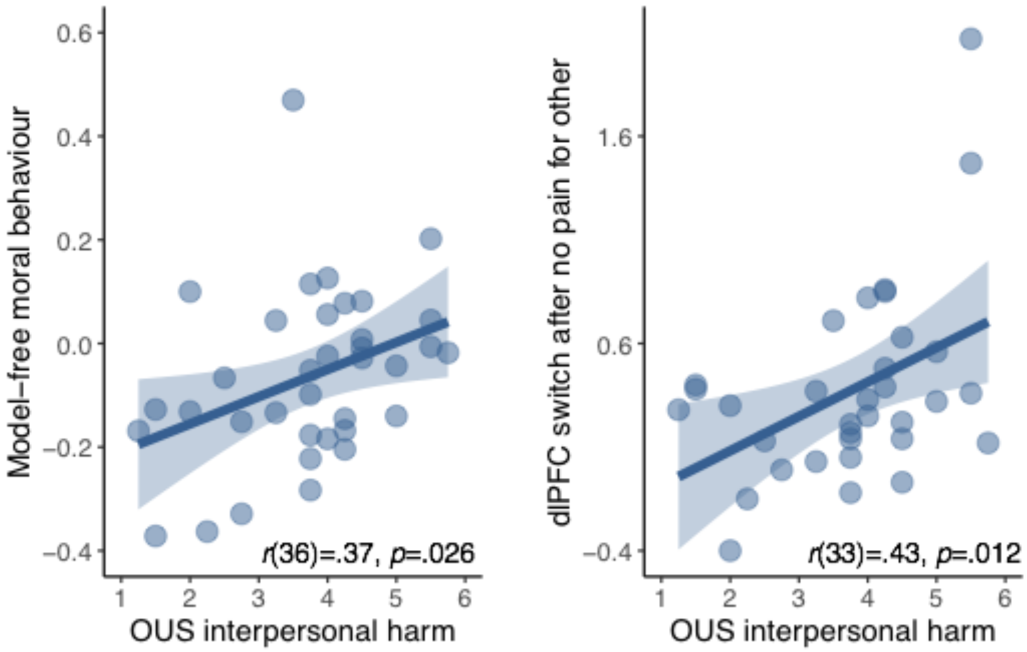

Fig. S6. Sensitivity to harm in moral judgments correlates with model-free moral behaviour and its neural correlates. (a) Partial correlation, controlling for harmful action sensitivity (as measured by the Harmful Action-Outcome Scale, Miller et al.[19]), between harmful outcome sensitivity and model-free moral behaviour. Model-free moral behaviour was the parameter estimate for the model free x recipient interaction that showed participants had a tendency to switch more after causing harm to other participant. Note the values are reversed on the y axis to depict that greater model-free behaviour is associated with greater harmful outcome sensitivity (b) Partial correlation controlling for harmful action sensitivity, between harmful outcome sensitivity and prediction errors of pain avoidance in the thalamus/caudate for Other compared to Self (c) Partial correlation controlling for harmful action sensitivity between harmful outcome sensitivity and parameter estimates extracted from the parametric regressor for stay vs switch after no pain for other in subgenual anterior cingulate cortex (sgACC). Note that values are reversed on the y axis such that a greater tendency to stay vs switch tracked in sgACC correlates with harmful outcome sensitivity.

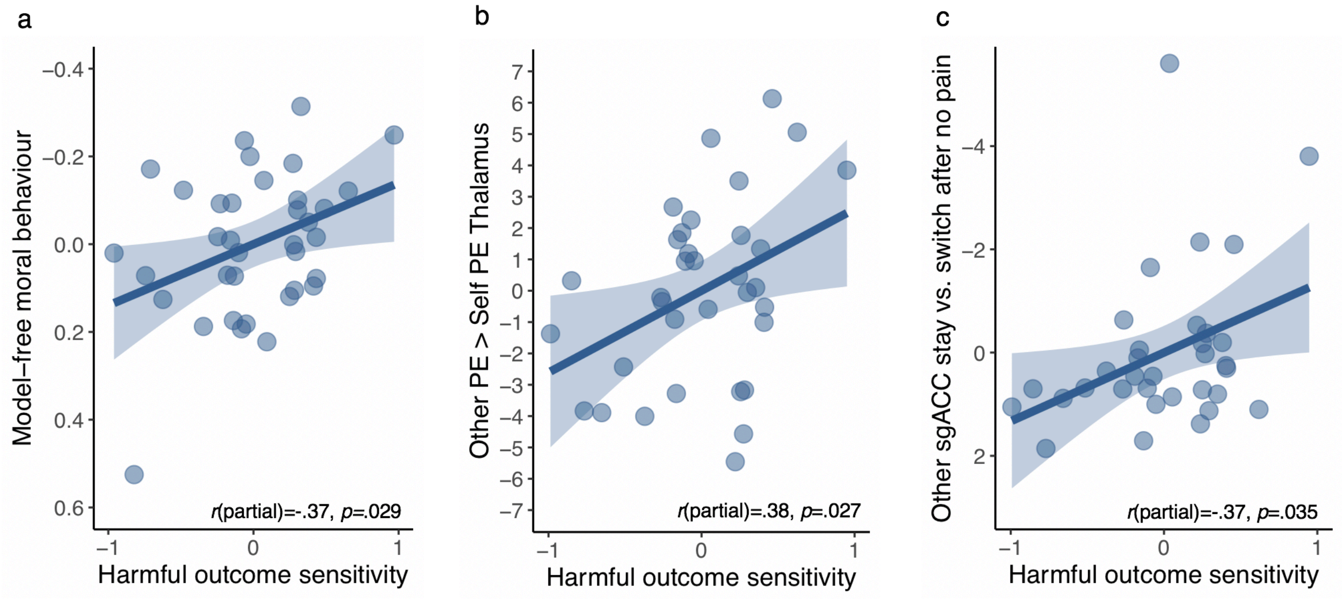

Fig. S7.

Correlation between parametric regressors in the model-based analyses. All correlations were below r<l0.26l, indicating that conditions could be appropriately estimated with independence from one another.

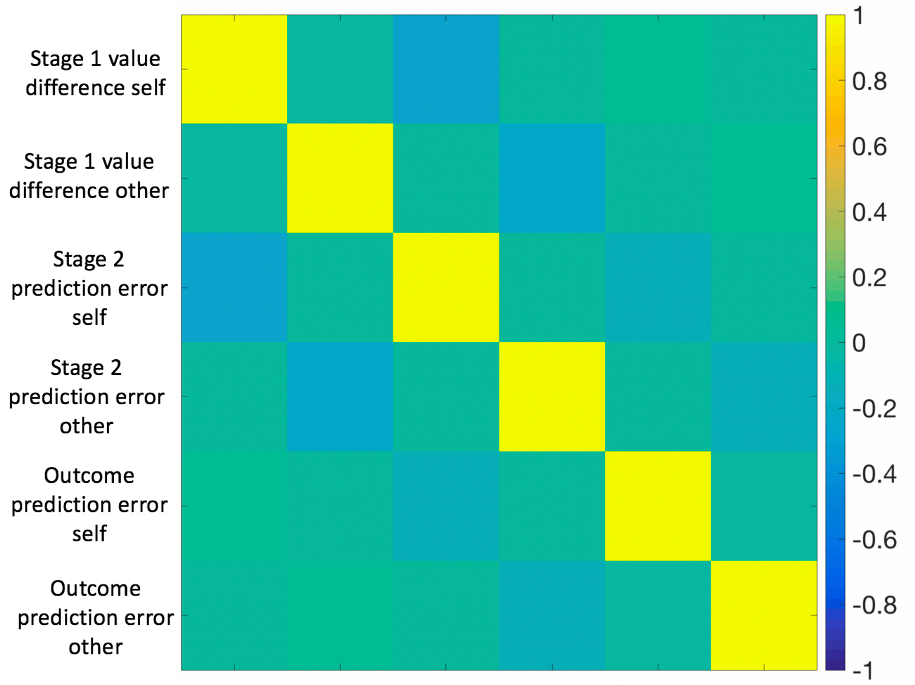

Fig. S8.

Correlation between parametric regressors in switch/stay analysis. All correlations were below r<|0.1|, indicating that conditions could be appropriately estimated with independence from one another.

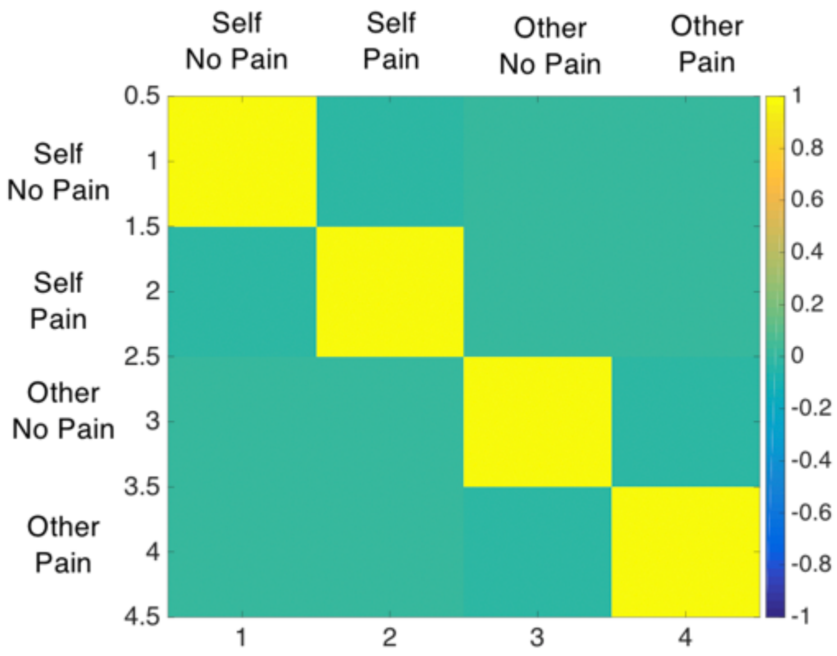

Table S1. **Model comparison self/other separately**

	Learning rate	Softmax temperature	Perseverance	Lambda	Model-free model-based weight	negLL	AIC	BIC	BICint	XP	Protected XP
SELF											
M5 (5-param)	α_{Pain}, α_{NoPain}	β	ρ	$\lambda=1$	ω	5342	11043	11567	11143	0.9999	0.9859
						ΔM5	ΔM5	ΔM5	ΔM5		
M4 (4-param)	α	β	ρ	$\lambda=1$	ω	113	154	49	136	0.0000	0.0035
M3 (5-param)	α	β	ρ	λ	ω	109	218	218	143	0.0001	0.0036
M2 (6-param)	α_{Stage1}, α_{Stage2}	β_{Stage1}, β_{Stage2}	ρ	$\lambda=1$	ω	93	257	362	190	0.0000	0.0035
M1 (7-param)	α_{Stage1}, α_{Stage2}	β_{Stage1}, β_{Stage2}	ρ	λ	ω	75	293	503	194	0.0000	0.0035
OTHER											
M5 (5-param)	α_{Pain}, α_{NoPain}	β	ρ	$\lambda=1$	ω	5318	10995	11520	11089	0.9588	0.6752
						ΔM5	ΔM5	ΔM5	ΔM5		
M4 (4-param)	α	β	ρ	$\lambda=1$	ω	109	146	41	153	0.0001	0.0748
M3 (5-param)	α	β	ρ	λ	ω	92	184	184	157	0	0.0747
M2 (6-param)	α_{Stage1}, α_{Stage2}	β_{Stage1}, β_{Stage2}	ρ	$\lambda=1$	ω	56	183	288	165	0	0.0748
M1 (7-param)	α_{Stage1}, α_{Stage2}	β_{Stage1}, β_{Stage2}	ρ	λ	ω	43	229	439	158	0.0411	0.1005

Separate model comparisons for self and other blocks. For both agents, a model with separate learning rates for no pain and pain (αPain, αNoPain), a single temperature parameter (β), a perseverance parameter (ρ) and a model-free/based weighting parameter (ω) performs best (model 5=M5).

Table S2 Model comparison both agents together

	Learning rate	Softmax temperature	Perse-verance	Lambda	Model-free model-based weight	negLL	AIC	BIC	BICint	XP
BOTH AGENTS										
M5 (5-param)	α_{Pain}, α_{NoPain}	β	ρ	λ=1	ω	10800	**21960**	**22609**	22175	0.8911
M6 (6-param)	α_{Pain}, α_{NoPain}	β	ρ	λ=1	ω_{Self}, ω_{Other}	10771	21974	22753	**22151**	0.1088
M7 (7-param)	α_{Pain}, α_{NoPain}	β	ρ_{Self}, ρ_{Other}	λ=1	ω_{Self}, ω_{Other}	**10754**	22012	22921	22225	0

Model comparison when fitting M5 and variants of it to the pooled data from self and other blocks. This does not support a strong conclusion. Nevertheless, the BICint slightly prefers M6 over the others. This model is identical to M5 but models separate model-free/model-based weights for self and other blocks (ω_{Self} and ω_{Other}). Crucially, there is a significant difference between the parameter estimates obtained for ω_{Self} and ω_{Other} providing further support for including both parameters.

Table S3 **Average parameter estimates self/other separately**

	M5		M4		M3		M2		M1	
	**Self**	**Other**	**Self**	**Other**	**Self**	**Other**	**Self**	**Other**	**Self**	**Other**
$\alpha/\alpha_{Pain}/\alpha_{Stage1}$	0.29	0.36	0.31	0.38	0.31	0.38	0.39	0.53	0.30	0.44
$\alpha_{NoPain}/\alpha_{Stage2}$	0.31	0.37					0.30	0.37	0.32	0.41
β/β_{Stage1}	4.55	3.85	4.25	3.42	4.24	3.59	5.06	4.72	6.29	5.02
β_{Stage2}							3.66	4.11	3.56	4.21
ρ	0.66	0.56	0.70	0.65	0.70	0.64	0.68	0.63	0.66	0.62
λ					0.95	0.81			0.53	0.54
ω	0.59	0.46	0.61	0.57	0.59	0.44	0.63	0.65	0.48	0.53

Average parameter estimates extracted for self/other blocks for the five different models. The winning model M5 showed differences in the perseverance parameter (ρ) and the model-free/based weighting parameter (ω) between agents.

Table S4. Average parameter estimates both agents together

	M5	M6	M7
α_{Pain}	**0.35**	**0.35**	**0.35**
α_{NoPain}	**0.36**	**0.35**	**0.35**
β	**3.68**	**3.81**	**3.81**
ρ/ρ_{Self}	**0.65**	**0.63**	**0.66**
ρ_{Other}			**0.57**
$\omega_{Both/Self}$	**0.53**	**0.55**	**0.56**
ω_{Other}		**0.45**	**0.44**

Average parameter estimates extracted for both agents fitted simultaneously for the three different models M5-M7. The winning model M6 contained one perseverance parameter (ρ) across both agents but separate model-free/based weighting parameters (ω) which differed significantly from each other.

Table S5. Whole brain analysis (*p*<.05 cluster correction after thresholding at p<.001).

Brain Region	BA	L/R	Peak voxel			k	t	z
**Main effect: Stage 1 value difference (negative)**								
Anterior cingulate		L	-6	24	44	625	5.69	5.11
		R	8	18	46		4.34	4.06
Inferior parietral		L	-34	-56	42	1830	5.08	4.65
		L	-48	-40	40		4.5	4.18
		L	-26	-64	46		4.37	4.08
Inferior parietal		R	38	-54	44	667	4.71	4.36
		R	42	-42	46		4.28	4.00
		R	50	-34	50		3.98	3.75
Middle Frontal gyrus		L	-34	46	22	312	4.69	4.34
		R	52	22	40	495	4.31	4.03
		R	48	32	22		4.01	3.78
		R	40	48	22		3.99	3.76
**Main effect: Stage 2 prediction error (negative)**								
Anterior cingulate		L	-6	10	52	906	5.34	4.85
		L	-4	18	44		5.15	4.71
		L	-20	4	62		3.58	3.41
**Main effect: Outcome prediction error (positive)**								
Ventral Striatum		R	10	12	-4	771	8.91	7.19
Ventral Striatum		L	-14	8	-10	5994	8.5	6.96
ext. Superior frontal gyrus			-18	26	60		6.3	5.55
ext. Inferior frontal gyrus			-46	4	26		6.06	5.38
Inferior parietal lobe		L	-48	-44	44	4740	7.99	6.66
			-46	-50	54		6.87	5.95
ext. Superior parietal lobe			-36	-68	52		5.92	5.28
Superior temporal gyrus		L	-56	-38	-16	774	6.45	5.66
			-62	-24	2		3.97	3.75
ext. Inferior temporal gyrus			-46	-58	-10		3.41	3.26
Precuneus		R	30	-66	38	2255	5.65	5.09
			44	-42	52		4.59	4.26
			48	-38	46		4.49	4.18
Middle occipital gyrus		R	32	-86	14		4.32	4.04
			30	-94	4		4.31	4.03

Table S6. Exceedance probabilities from the Bayesian Model Selection analysis comparing a prediction error model (PE, model 1) to an outcome only model (M2), shown at different peak co-ordinates in thalamus and ventral striatum. There was anatomical specificity and both models best explained the BOLD signal in different sub-peaks of the two areas we identified in our main analysis (GLM1) suggesting responses in different locations can be interpreted as reflecting the tracking of prediction errors or the tracking of outcomes.

Brain area	Peak voxel			M1 (PE)	M2 (outcome)
Thalamus	10	-4	4	0.97	0.03
Thalamus	16	-18	0	0.00	1.00
Thalamus	12	-2	4	0.43	0.57
Ventral Striatum	10	12	-4	0.01	0.99
Ventral Striatum	-16	6	-10	0.91	0.09

SI References

1. Akam, T., Costa, R. & Dayan, P. Simple Plans or Sophisticated Habits? State, Transition and Learning Interactions in the Two-Step Task. *PLOS Comput. Biol.* **11**, e1004648 (2015).
2. Kool, W., Cushman, F. A. & Gershman, S. J. When does model-based control pay off? *PLoS Comput. Biol.* **12**, e1005090 (2016).
3. Doll, B. B., Duncan, K. D., Simon, D. A., Shohamy, D. & Daw, N. D. Model-based choices involve prospective neural activity. *Nat. Neurosci.* **18**, 767 (2015).
4. Daw, N. D., Gershman, S. J., Seymour, B., Dayan, P. & Dolan, R. J. Model-based influences on humans' choices and striatal prediction errors. *Neuron* **69**, 1204–1215 (2011).
5. Wittmann, M. K., Lockwood, P. L. & Rushworth, M. F. S. Neural Mechanisms of Social Cognition in Primates. *Annu. Rev. Neurosci.* (2018) doi:10.1146/annurev-neuro-080317-061450.
6. Ruff, C. C. & Fehr, E. The neurobiology of rewards and values in social decision making. *Nat. Rev. Neurosci.* **15**, 549–562 (2014).
7. Lockwood, P. L. *et al.* Prosocial apathy for helping others when effort is required. *Nat. Hum. Behav.* **1**, 0131 (2017).
8. Evans, A. M., Dillon, K. D. & Rand, D. G. Fast but not intuitive, slow but not reflective: Decision conflict drives reaction times in social dilemmas. *J. Exp. Psychol. Gen.* **144**, 951 (2015).
9. Bear, A., Kagan, A. & Rand, D. G. Co-evolution of cooperation and cognition: the impact of imperfect deliberation and context-sensitive intuition. *Proc. R. Soc. B Biol. Sci.* **284**, 20162326 (2017).
10. Konovalov, A. & Krajbich, I. Gaze data reveal distinct choice processes underlying model-based and model-free reinforcement learning. *Nat. Commun.* **7**, 12438 (2016).
11. Cogliati Dezza, I., Cleeremans, A. & Alexander, W. Should we control? The interplay between cognitive control and information integration in the resolution of the exploration-exploitation dilemma. *J. Exp. Psychol. Gen.* **148**, 977–993 (2019).
12. Otto, A. R., Raio, C. M., Chiang, A., Phelps, E. A. & Daw, N. D. Working-memory capacity protects model-based learning from stress. *Proc. Natl. Acad. Sci.* **110**, 20941–20946 (2013).
13. Huys, Q. J. M. *et al.* Bonsai trees in your head: how the pavlovian system sculpts goal-directed choices by pruning decision trees. *PLoS Comput. Biol.* **8**, e1002410 (2012).

14. Huys, Q. J. M. *et al.* Disentangling the Roles of Approach, Activation and Valence in Instrumental and Pavlovian Responding. *PLOS Comput. Biol.* **7**, e1002028 (2011).

15. Mackay, D. Model comparison and Occams Razor. *Inf. Theory Inference Learn. Algorithms* 343–355 (2003).

16. Palminteri, S., Wyart, V. & Koechlin, E. The Importance of Falsification in Computational Cognitive Modeling. *Trends Cogn. Sci.* **21**, 425–433 (2017).

17. Lockwood, P. L. & Klein-Flügge, M. C. Computational modelling of social cognition and behaviour—a reinforcement learning primer. *Soc. Cogn. Affect. Neurosci.* doi:10.1093/scan/nsaa040.

18. Kahane, G. *et al.* Beyond sacrificial harm: A two-dimensional model of utilitarian psychology. (2017).

19. Miller, R. M., Hannikainen, I. A. & Cushman, F. A. Bad actions or bad outcomes? Differentiating affective contributions to the moral condemnation of harm. *Emotion* **14**, 573 (2014).
